# Polygenic Risk Scores for Subtyping of Schizophrenia

**DOI:** 10.1155/2020/1638403

**Published:** 2020-07-23

**Authors:** Jingchun Chen, Travis Mize, Jain-Shing Wu, Elliot Hong, Vishwajit Nimgaonkar, Kenneth S. Kendler, Daniel Allen, Edwin Oh, Alison Netski, Xiangning Chen

**Affiliations:** ^1^Nevada Institute of Personalized Medicine, University of Nevada, Las Vegas, NV 89154, USA; ^2^Department of Psychology, University of Nevada, Las Vegas, NV 89154, USA; ^3^Department of Psychiatry, University of Maryland, Baltimore, MD 21228, USA; ^4^Department of Psychiatry, University of Pittsburgh, Pittsburgh, PA 15213, USA; ^5^Department of Psychiatry, Virginia Commonwealth University, Richmond, VA 23298, USA; ^6^Department of Psychiatry and Behavioral Health, University of Nevada, Las Vegas, School of Medicine, NV 89102, USA; ^7^410 AI, LLC, Germantown, MD 20876, USA

## Abstract

Schizophrenia is a complex disorder with many comorbid conditions. In this study, we used polygenic risk scores (PRSs) from schizophrenia and comorbid traits to explore consistent cluster structure in schizophrenia patients. With 10 comorbid traits, we found a stable 4-cluster structure in two datasets (MGS and SSCCS). When the same traits and parameters were applied for the patients in a clinical trial of antipsychotics, the CATIE study, a 5-cluster structure was observed. One of the 4 clusters found in the MGS and SSCCS was further split into two clusters in CATIE, while the other 3 clusters remained unchanged. For the 5 CATIE clusters, we evaluated their association with the changes of clinical symptoms, neurocognitive functions, and laboratory tests between the enrollment baseline and the end of Phase I trial. Class I was found responsive to treatment, with significant reduction for the total, positive, and negative symptoms (*p* = 0.0001, 0.0099, and 0.0028, respectively), and improvement for cognitive functions (VIGILANCE, *p* = 0.0099; PROCESSING SPEED, *p* = 0.0006; WORKING MEMORY, *p* = 0.0023; and REASONING, *p* = 0.0015). Class II had modest reduction of positive symptoms (*p* = 0.0492) and better PROCESSING SPEED (*p* = 0.0071). Class IV had a specific reduction of negative symptoms (*p* = 0.0111) and modest cognitive improvement for all tested domains. Interestingly, Class IV was also associated with decreased lymphocyte counts and increased neutrophil counts, an indication of ongoing inflammation or immune dysfunction. In contrast, Classes III and V showed no symptom reduction but a higher level of phosphorus. Overall, our results suggest that PRSs from schizophrenia and comorbid traits can be utilized to classify patients into subtypes with distinctive clinical features. This genetic susceptibility based subtyping may be useful to facilitate more effective treatment and outcome prediction.

## 1. Introduction

Schizophrenia is a severe mental disorder with heterogeneous genetic architecture and clinical presentation [1–4]. As a heritable disorder, schizophrenia has an estimated heritability of about 80% [5], and genome-wide association studies (GWASs) have identified more than 100 loci [6–9]. Clinically, schizophrenia patients present positive and negative symptoms and cognitive deficits [3, 4, 10–12]. Furthermore, symptoms presented in individuals may change as the disease progresses [2]. All these impose great challenges for both genetic and clinical studies, hindering effective treatment and therapy of this disorder.

Subtyping is an effective approach to reduce heterogeneity, and it has been applied to complex diseases such as breast cancer [13, 14] and stroke [15, 16]. However, subtyping psychiatric disorders are challenging. Specific to schizophrenia, attempts to subtype with clinical symptoms [4, 17–20], neurocognitive functions [12, 21–26], age of onset [27, 28], treatment responses [29–31], and specific genetic risk factors [24, 32–35], had been reported in the literature. A 5-subtype classification based on clinical symptoms was enacted in the Diagnostic and Statistical Manual of Mental Disorders, 4^th^ Edition (DSM IV) [19]. However, most of these subtyping systems lack biological underpinning, measurement objectivity, or systematic perspectives. As a result, they have not been broadly implemented in clinical practice and have not demonstrated utility in the patient care. For these reasons, the 5-subtype classification was removed from DSM V. Given the challenges and potential benefits, it is important to consider whether we can develop a data-driven method to subtype schizophrenia so that the resulting subtypes can be used to guide clinical practice and have a more homogeneous biological mechanism.

Recent findings from large scale GWASs [36] indicated that pleiotropy is pervasive [37, 38] and that comorbid traits share some genetic liability [37, 39]. These findings present us with such an opportunity. We reasoned that schizophrenia is comorbid with many other mental disorders [40, 41] and physical diseases [42] and that many comorbid conditions share genetic liability, genetic factors identified for both schizophrenia and the diseases and traits comorbid with schizophrenia may be used as effective classifiers to subtype schizophrenia. Since these diseases and traits share only partial genetic liability with schizophrenia, i.e., some schizophrenia patients share genetic liability with one condition, while others share liability with a different condition, collectively, these conditions could segregate schizophrenia patients into different classes or subtypes. Furthermore, this differential sharing of genetic liability implies that the resulting subtypes have distinctive underlying biology, and therefore, more targeted and subtype-specific treatments may be imposed for better outcomes. In this study, we hypothesize that the partial sharing of genetic liability can be used to classify schizophrenia into distinct subtypes with different dimensions of genetic risk, and the resulting subtypes may reveal different pathophysiology. An expected outcome of this hypothesis is that these genetically informed subtypes have a unique underlying genetic architecture and underpinning biology that can be verified objectively and accurately by clinical and laboratory tests.

**Figure 1:**
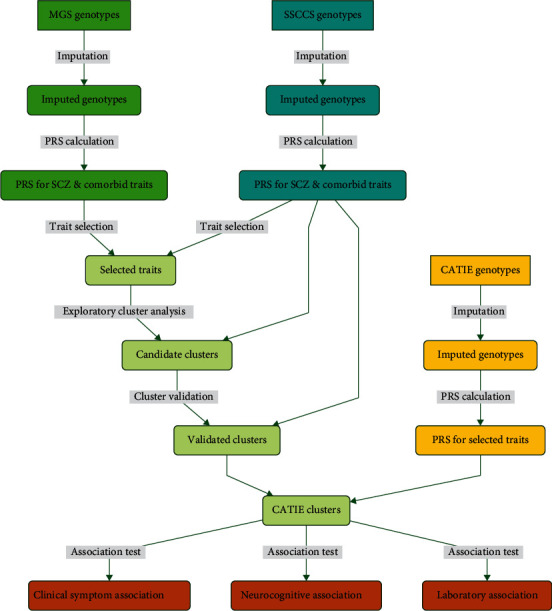
A flowchart illustrating the design of the study. The MGS and SSCCS datasets were used to screen for comorbid traits and establish stable cluster structure. The CATIE dataset was used to verify the derived clusters or classes that were further tested for their association with clinical symptoms, neurocognitive functions, and laboratory tests.

In this report, we describe our study to test the hypothesis. We started with the selection of traits that are genetically related to schizophrenia and estimated their polygenic risk scores (PRSs) in three independent datasets: the Molecular Genetics of Schizophrenia (MGS), the Swedish Schizophrenia Case-Control Study (SSCCS), and the Clinical Antipsychotic Trials for Intervention Effectiveness (CATIE) datasets. Next, we used hierarchical cluster algorithms to group subjects by their shared genetic liability in the MGS dataset. Then, we verified the cluster structure with the SSCCS and the CATIE datasets. Finally, we validated the resulting subtypes with clinical, neurocognitive, and laboratory tests in the CATIE dataset. The overall study design is shown in Figure 1. Our results suggest that it is possible to classify schizophrenia patients based on the partially shared genetic liability with other comorbid conditions, highlighting the potential in the genetically based treatment and intervention for schizophrenia.

## 2. Results

### 2.1. Selection for Traits Genetically Related to Schizophrenia

We started our study with PubMed search using keywords “schizophrenia”, “comorbidity”, and “genome-wide association study”, or “GWAS” and cross-linked the traits with data at GWAS catalog repository website (https://www.ebi.ac.uk/gwas/) and other sources. As a result, we obtained GWAS summary statistics for 25 diseases and traits (Table S1). Using the markers with *p* ≤ 0.05 in both schizophrenia and the candidate traits, PRSs from these traits were calculated for the subjects in MGS [43], SSCCS [7], and CATIE [44, 45] datasets for this study. We conducted linear regression to evaluate the genetic relationships between schizophrenia diagnosis and the PRSs calculated from these candidate traits. Only those traits showing suggestive association signals (*p* ≤ 0.15) and the same direction of effect in both MGS and SSCCS datasets were selected for inclusion in our subtype classification analyses. As shown in Table 1, 10 traits showed a consistent correlation with schizophrenia. As expected, bipolar disorder (BIP) [44], cannabis dependence (cannabis) [45], and ever smoker vs never smoker (evrSmk) [46] showed a positive correlation with schizophrenia, whereas subjective wellbeing (SWB) [47] and verbal and numeric reasoning (VNR) [48] were negatively correlated with schizophrenia. Surprisingly, working memory (MEM) [48], Neoopenness (OPEN) [49], and years of school attended (YoS) [50] were also positively correlated with schizophrenia.

### 2.2. Cluster Analyses

**Figure 2:**
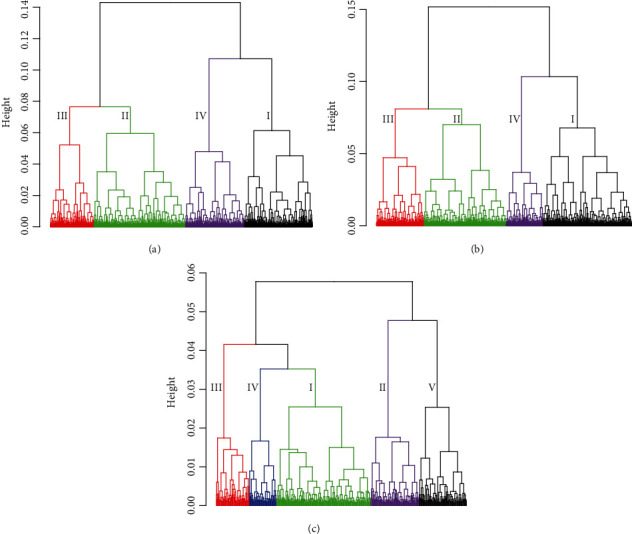
Cluster dendrograms based on 10 traits genetically related to schizophrenia. Cluster dendrograms of the MGS (a), SSCCS (b), and CATIE (c) datasets. The Roman numbers indicated the classes used in our association analyses. For the MGS and SSCCS datasets, subjects were classified into 4 classes, and for the CATIE dataset, subjects were classified into 5 classes. The classes were color-coded.

Based on the analyses of genetic relationships, the PRSs of the 10 selected traits along with those of schizophrenia were used in our cluster analyses. Our objective here was to find a stable and consistent cluster structure as the base for our subtyping analyses. Towards this goal, we first used the R package “NbClust” [51] to explore the appropriate number of clusters for the MGS dataset and then validated the structure in the SSCCS dataset. We found that the 4-cluster solution was the one with the most endorsements (11 out of 20 indices) for cluster assessment (Figure 2(a)) for the MGS dataset. When we used the same parameters to verify the cluster structure with the independent SSCCS dataset, we found that the 4-cluster solution also had the most endorsements (7 out of 20) (Figure 2(b)). Based on these analyses, we concluded that the 4-cluster solution was a stable and consistent cluster structure for schizophrenia patients when assessed with PRSs from the 10 selected candidate traits.

With the same parameters from the analyses of both the MGS and SSCCS datasets, we ran the analyses for another independent CATIE dataset A 3-cluster solution had the most endorsements (6 out of 20). However, further examination of the dendrograms indicated that cluster 1 could be divided further into three groups for CATIE dataset (the red, blue, and green clusters in Figure 2(c)). Therefore, we decided to use a 5-cluster solution for the CATIE dataset. Of note, if Classes I and IV in the CATIE were merged, it would result in a 4-cluster structure similar to that observed from the MGS and SSCCS datasets.

As mentioned earlier, the central goal of this study was to evaluate whether schizophrenia had a stable and consistent subtype structure with genetic and biological underpinning. The PRSs-based cluster analyses seemed supporting this notion. In the sections below, we intended to test this hypothesis by examining class membership association with a variety of clinical, neurological, and laboratory tests.

### 2.3. Subtype Class Association with Clinical Symptoms and Treatment Outcomes

**Figure 3:**
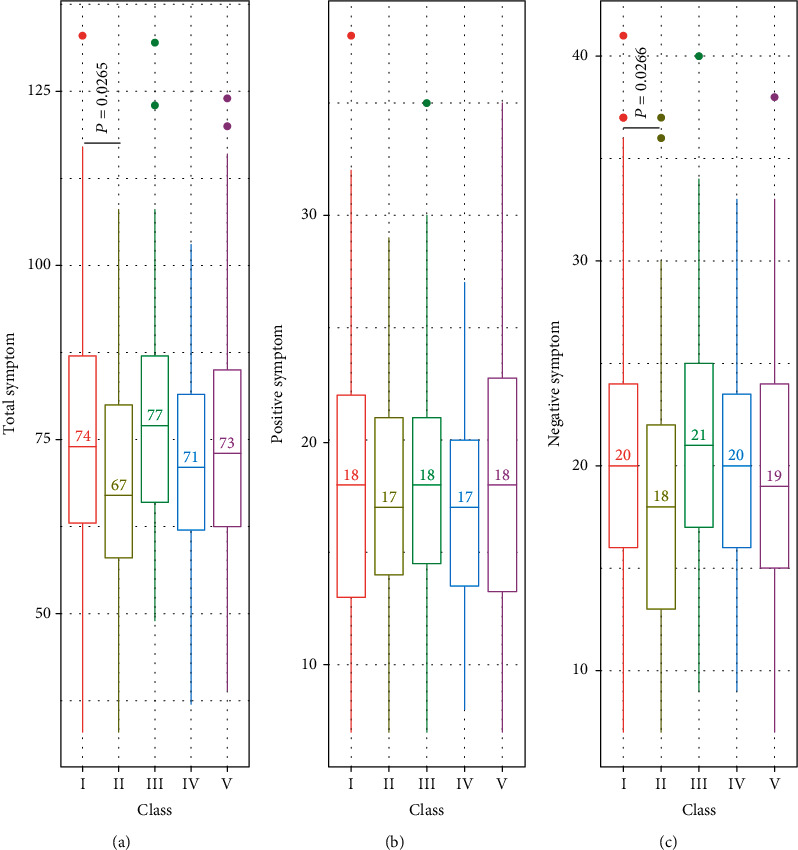
Boxplot of baseline clinical symptoms by class. Class II had statistically lower total symptom counts and negative symptoms. The numbers shown were medians of symptom counts for the classes.

To examine whether the classes had any pattern in clinical symptoms, we analyzed the association of class membership with clinical symptoms using the CATIE study, a multivisit clinical trial of antipsychotics treatment on schizophrenia that last up to 18 months in the end Phase I trial [52, 53]. During the trial, clinical evaluations, psychological and behavioral assessments, and laboratory tests were performed to evaluate their responses of antipsychotics the entrance (baseline assessments), each follow-up visit, and the end of the trial. To evaluate class membership association with clinical symptoms, we used the clinical data at baseline and at the end of the 18-month Phase I trial. We first analyzed the total, positive, and negative symptoms at the baseline across the classes, and found that Class II had lower total symptoms and negative symptoms at baseline as compared to Class I, which was used as reference (Figure 3).

Next, we examined if any classes were associated with treatment outcomes as defined by the differences of symptom counts between baseline and at the end of the 18-month Phase I trial. When all subjects were analyzed together, the total, positive, and negative symptoms were all significantly reduced (Table 2). The total symptom counts reduced by 4.17 (95% CI 2.59–5.75, *p* = 3.5E-07), the reduction of positive and negative symptoms was 1.18 (95% CI 0.63–1.73, *p* = 2.78E-05) and 0.93 (95% CI 0.39–1.48, *p* = 7.94E-04), respectively. This suggested that the antipsychotic treatment during Phase I trial had positive outcomes for the CATIE patients as a whole. When the classes were analyzed separately, Class I had the most significant improvements in symptom counts for the total (mean diff = 5.47, 95% CI 2.76–8.19, *p* = 1.02E-04), positive (mean diff = 1.26, 95% CI 0.31–2.22, *p* = 9.90E-03), and negative (mean diff = 1.36, 95% CI 0.47–2.25, *p* = 2.81E-03) symptoms by the end of Phase I trial. Class I results remained significant after Bonferroni correction (3 tests for each category). Class II had lower total symptoms (mean diff = 3.90, 95% CI 0.78–7.03, mean diff = 2.18), and Class IV had lower negative symptoms (mean diff 2.18, 95% CI 0.53–3.82, *p* = 0.0111). In clear contrast, Classes III and V had no significant changes for the total, positive, and negative symptoms.

### 2.4. Subtype Class Association with Neurocognitive Functions

We examined the changes of neurocognitive functions by comparing the data from baseline and Visit 6, which was approximately 170 days into Phase I trial (mean = 173.40, *s*.*e*. = 0.46). We used the data from Visit 6, because it gave us the largest sample size for neurocognitive function analyses. As shown in Table 3, Phase I treatment did have a significant improvement for cognitive functions when all subjects were analyzed together (VIGILANCE, mean diff = −0.30, 95% CI −0.40–−0.19, *p* = 3.0E-07; PROCESSING SPEED, mean diff = −0.24, 95% CI −0.32–−0.17, *p* = 6.8E-10; WORKING MEMORY, mean diff = −0.20, 95% CI −0.29–−0.11, *p* = 2.8E-05; and REASONING, mean diff = −0.27, 95% CI −0.38–−0.17, *p* = 5.8E-07). When the classes or subtypes were analyzed separately, Class I showed significant improvements for all cognitive domains (VIGILANCE, *p* = 5.8E-07, 95% CI −0.40–−0.06, *p* = 0.0099; PROCESSING SPEED, mean diff = −0.20, 95% CI −0.31–−0.09, *p* = 0.0006; WORKING MEMORY, mean diff = −0.21, 95% CI −0.34–−0.08, *p* = 0.0023; and REASONING, mean diff = −0.28, 95% CI −0.45–−0.11, *p* = 0.0015), which all passed Bonferroni correction (4 tests for each domain). Class II had an improvement in PROCESSING SPEED only, and Class III had improvement for VIGILANCE only. Class V had improvements for VIGILANCE and PROCESSING SPEED. Class IV showed a tendency of improvement in all domains but only REASONING survived multiple testing correction.

### 2.5. Subtype Class Association with Clinical Laboratory Tests

The purpose to include these tests was to find whether there were any laboratory tests associated with our subtype classification. Using the baseline data to compare to Class I, we found that patients from Class II had lower levels of bilirubin effect size = −0.09, *p* = 0.058) and uric acid (effect size = −0.46, *p* = 0.0243) and that patients from Class V had a higher level of prolactin (effect size = 9.74, *p* = 0.0365) (Table S2).

Next, we conducted paired *t*-tests to compare whether any of the laboratory tests changed between the baseline and at the end of the Phase I trial. These statistical tests would provide us information on how Phase I treatment impacted the patients and how they related to our subtype classification. When all subjects were analyzed together at the end of the Phase I trial, patients had a higher level of calcium (mean diff = 0.08, 95% CI 0.04–0.12, *p* = 0.0003) and phosphorus (mean diff = 0.10, 95% CI 0.04–0.17, *p* = 0.0029), as compared to their baseline measurement (Table 4). When each class was analyzed separately, Class I patients had lower HDL than that measured at baseline (mean diff = −2.25, 95% CI −3.70–−0.79, *p* = 0.0027). Both Classes III and V had higher level of phosphorus (mean diff = 0.19 and 0.17; 95% CI 0.02–0.37 and 0.02–0.32; *p* = 0.0282 and 0.0276, respectively). In addition, Class V also had higher levels of HDL and calcium (mean diff = 2.22 and 0.14; 95% CI 0.30–4.13 and 0.04–0.24; *p* = 0.0239 and 0.0084, respectively). The results from Classes III and V suggested the patients from these two groups might have metabolic syndrome, a possible side effect from antipsychotics. Furthermore, Class IV had significant changes for cell counts of decreased lymphocytes (mean diff = −3.12, 95% CI −5.32–−0.92, *p* = 0.0065) and increased neutrophils (mean diff = 3.04, 95% CI 0.47–5.61, *p* = 0.0214). Therefore, neutrophil to lymphocyte ratio (NLR) was increased in this group, suggesting those patients were under inflammation or linked to immune dysfunctions.

### 2.6. Post Hoc Analyses of Other Clinical Features and Laboratory Tests

To explore whether these subtypes had other specific clinical features documented in the CATIE dataset, we conducted post hoc analyses with the data of the structured clinical interview for DSM-IV axis I disorders (SCID), a clinical global impression (CGI), and trial discontinuation—the major outcome measures for the CATIE study. From the SCID data, we found that compared to Class I, Class IV had older age when first prescribed antipsychotic medicine (effect size = 2.70, *p* = 0.0363) and was more likely to have a family history of mental illness (effect size = 0.69, *p* = 0.0331) (Table S3). From CGI data, Class II was more likely to use tobacco products in the last 3 months (effect size = 0.64, *p* = 0.0219) (Table S4). From the data of medication discontinuation, we found that Class IV patients were less likely to discontinue their clinical trials even there was no treatment effect (effect size = −0.87, *p* = 0.0299) (Table S5). While the results from post hoc analyses were suggestive and could not survive multiple test correction, they were consistent with the results from the analyses of clinical symptoms and neurocognitive functions.

## 3. Conclusion and Discussion

In this study, we used the differentially shared genetic liability between schizophrenia and comorbid traits to classify and subtype schizophrenia. Using the PRSs from 10 comorbid traits, we classified the 435 patients in the CATIE study into 5 classes. As summarized in Table 5, patients from Class I had the best responses to antipsychotic therapy for symptom reduction at the end of Phase I trial and improvements for cognitive functions at Visit 6. This class could be considered treatment responsive group. After treatment, Class II showed signs of symptom reduction for the total symptoms and positive symptoms and improvement for PROCESSING SPEED. Class IV was uniquely responsive to treatment for negative symptoms and tended to improve for all cognitive domains. Interestingly, Class IV had a significant decrease in cell count for lymphocytes but an increase for neutrophils at the end of Phase I trial, suggesting that the patients in Class IV may have an ongoing inflammation condition or immune dysfunction. In contrast, patients in both Classes III and V did not have symptom reduction by the end of Phase I trial but only partial improvements for cognitive function. Patients in Class III had a higher level of phosphorus by the end of Phase I. Similarly, patients in Class V also had a higher level of phosphorus, while they had additional higher levels of HDL and calcium. Based on their treatment outcomes, Classes III and V could be considered treatment-resistant. The changes in laboratory tests from Classes III and V were indicative of the development of metabolic syndrome-related to antipsychotic drug treatment. A significant increase in phosphorus levels has been linked to some schizophrenia clinical subtypes [54]. The side effects may interfere with the treatment compliance that causes the treatment-resistant. Overall, these five classes showed some unique patterns with regard to clinical symptoms, cognitive functions, treatment outcomes, laboratory tests, and other clinical features. These results were consistent with the hypothesis that partially shared genetic liability between schizophrenia and comorbid traits could be used to classify patients into subtypes with distinct underlying biology.

As mentioned in the introduction, although there were attempts to subtyping schizophrenia using varying approaches, our study is the first to combine genetic liability to schizophrenia and other comorbid conditions for subtype classification. Our approach differs from previous studies in two aspects. First, our approach is systematic and data-driven. We used genome-wide association data to evaluate genetic liability to schizophrenia. More and more evidence suggests that the genetic architecture of schizophrenia is complex, and causal variants in patients may differ substantially. Profiling genetic liability across the entire genome could reveal the difference between individual patients. Grouping patients based on their genetic liability, i.e., subtyping, could reduce the heterogeneity within the group, leading to a better understanding of the underlying biology and new treatment strategy. The availability of genome-wide genetic data makes this data-driven approach feasible. To our knowledge, no previous study uses genome-wide data to address this issue. While there is room for improvement, our exploratory results are promising and encouraging. Second, our approach integrates partially overlapped genetic liability from comorbid conditions to subtype schizophrenia patients. Previous studies, regardless of the use of clinical symptoms, neurocognitive functions, or treatment outcomes, it is schizophrenia-centric, comorbid conditions rarely contribute to the subtyping. This is partially due to the difficulty to differentiate the same symptoms obtained from schizophrenia and comorbid conditions. With the use of genome-wide genetic profiling, i.e., PRSs, from comorbid conditions, we could distinguish to what extent that the bipolar disorder and major depression factors contribute to schizophrenia. When multiple comorbid conditions are incorporated, the difference between individual patients would be more distinctive, leading to better separation of subgroups or subtypes. The finding that different classes have a distinct association with clinical symptoms and laboratory tests is supportive of this notion.

Our study has important implications. First, our classification is associated with treatment outcomes. Classes I and IV had better outcomes as measured by symptom count reduction and improvements in 4 aspects of cognitive function. Class IV was also less likely to discontinue medication even though the antipsychotic drugs showed no effects. In contrast, Classes III and V had no reduction of symptoms but only partial improvements in cognitive function by the end of the Phase I trial. This contrast in treatment outcomes is of clinical importance, especially for prognostic prediction. Second, our classification is associated with specific laboratory tests. Clinical laboratory tests are a cornerstone of modern medicine; they constitute the basis for the diagnosis and treatment for many physical diseases. Our analyses of the CATIE dataset indicated that Class IV was associated with the cell count changes of lymphocytes and neutrophils between the baseline and the end of Phase I trial. As we all know, both lymphocytes and neutrophils are important parts of the immune system. Lymphocytes are responsible for antigen recognition and antibody production, while neutrophils respond to inflammation and kill invaded microorganisms and damaged cells. Since the connection between schizophrenia and the immune system is well documented in the literature, this specific association of Class IV and the immune cells suggests unique underlying pathophysiology. On the other hand, an elevated NLR is widely considered as an indicator of inflammation, because the physiological response of leukocytes to inflammation often leads to higher neutrophils and lower lymphocytes in the body [55]. Indeed, NLR has been reported to be related to the different stages of schizophrenia, supporting inflammation or immune hypothesis in schizophrenia [56]. For Classes III and V, the elevated phosphorus accompanying with other laboratory test changes such as HDL and electrolytes indicate that those patients may have ongoing metabolic syndrome. The specific association between these laboratory tests and subtype classes implies distinctive underlying biology. These tests may be used as biomarkers for subtyping and treatment evaluation. Third, the features associated with the subtype classes provide new insights for our understanding of disease pathophysiology and new strategies for treatment. For example, Class IV had a significant reduction of negative symptoms, but not a reduction of positive symptoms after Phase I trial. An elevated NLR from a simple complete blood count (CBC) often reveals an ongoing inflammation. This would explain why positive symptoms did not have a reduction among those patients, for whom treatment including adjunctive use of nonsteroid anti-inflammatory drugs would be beneficial, as previously reported [57]. Class IV was also associated with older age when the patients were first prescribed antipsychotic medicine and a family history of mental illness. This subtype also had an improvement for reasoning after Phase I. When all this information is considered together, some intriguing questions emerge. Is there a relationship between positive symptoms and inflammation or immune dysfunction? Would a treatment strategy that combines antipsychotics and anti-inflammatory drugs or immune therapy perform better for Class IV patients than a standard antipsychotics treatment? Do the age of onset (inferred by the age of the first prescription of antipsychotics) and family history of mental illness relate to treatment outcomes and reasoning? For Class V, due to its link to the metabolic syndrome, would simultaneous treatments with antipsychotics and drugs for the metabolic syndrome, or avoidance of antipsychotic drugs that increase the risk for developing metabolic syndrome, lead to better outcomes? Is an elevated level of phosphorus more related to treatment-resistant? Further investigation of these questions could help our understanding of subtype-specific mechanisms and provide the basis for the development of subtype-specific treatments.

Our study has some limitations. First, although we found class-specific associations with symptom counts, treatment outcomes, cognitive improvements, and clinical laboratory tests in CATIE patients, we could not validate these findings with independent samples. This is largely due to the limitation of available data. Further study with appropriate data is necessary to confirm our results. Second, although we screened 25 comorbid traits, our screening was by no means exhaustive. When more traits are screened, more candidate traits could be included and different cluster structures may be found. It may require multiple iterations and additional comorbid traits to reach an optimal cluster structure. Third, the demographics and the clinical data from the MGS and SSCCS datasets are currently not publicly available. Based on the diagnosis only, we may have missed some hidden confounder factors when we compare the CATIE sample with those two samples.

In summary, this study provides the first demonstration that differentially shared genetic variants between comorbid traits can be utilized to subtype schizophrenia into classes associated with specific clinical features, treatment outcomes, cognitive improvements, and laboratory tests. Patients in the CATIE study were classified into 5 classes. Classes I and IV had varying levels of treatment responses as measured by symptom reduction and had cognitive improvements for all measured domains, with Class I having better outcomes. They would be the treatment responsive group. Classes III and V had no symptom reductions but with only partial cognitive improvement after Phase I trial. They could be considered the treatment-resistant group. Using laboratory tests as a measure, Class IV would have ongoing inflammation or immune dysfunction, and Classes III and V may have metabolic syndrome. This classification may be translated into a class-specific treatment strategy. Our study is of clinical importance and mechanistic significance; it provides the evidence that data-driven subtyping, biology-based, and subtype-specific treatment of schizophrenia may be accomplishable. However, due to the limitation of data availability mentioned above, our findings need further validation.

## 4. Methods

### 4.1. Genotype Datasets

We applied for and obtained the genotype and clinical data for the MGS [43], SSCCS [7], and the CATIE [52, 53] datasets from NIMH Genetics Repository (https://www.nimhgenetics.org/). Both the MGS and SSCCS datasets were large genetic studies of schizophrenia using a case-control design. The CATIE study was a clinical trial to evaluate the efficacy of antipsychotics treatment on schizophrenia. The MGS and CATIE datasets were genotyped with Affymetrix 6.0 microarray with about 906,600 SNPs. The SSCCS was typed with Illumina OmniExpression array with 713,599 SNPs. In order to have the same markers across the MGS, SSCCS, and CATIE datasets, we used the IMPUTE2 [58] to impute the downloaded genotypes with the 1000 Genome haplotypes as reference. Markers with the INFO value <0.4 were filtered out. Details of imputation were described previously [59].

The genetic scores were used for the evaluation of the genetic relationships between schizophrenia and comorbid traits. Imputed genotype data were used for PRS calculation from MGS, SSCCS, and CATIE datasets including subjects (cases and controls). All subjects used were of European ancestry.

For cluster analyses or subtyping, only cases (affected schizophrenia individuals) were used as we are only interested in the subtype for the patients. The MGS dataset had 2,681 cases, SSCCS had 2,895 cases, and CATIE had 435 cases. Overall, MGS and SSCCS were used to discover traits comorbid with schizophrenia and explore cluster structure. The CATIE was used to verify the cluster structure and validate subtypes with clinical symptoms, treatment outcomes, neurocognitive functions, and laboratory tests (Figure 1).

### 4.2. Comorbid Trait Selection and Genetic Relationship with Schizophrenia

Based on the literature search by keywords “schizophrenia”, “comorbidity”, and “genome-wide association studies”, or “GWAS” from https://pubmed.ncbi.nlm.nih.gov/, we selected 25 psychiatric and physical diseases/traits that are comorbid with schizophrenia. We then downloaded the GWAS summary statistics from the GWAS catalog website (various sources). A total of 25 traits chosen were chosen (Table S1). We calculated PRSs for each specific trait [60] using markers with *p* values ≤0.05 in both schizophrenia and the candidate traits, which had been shown to optimally capture phenotypic variance in a previous study [8]. Scores were weighted by the logarithm of the odds ratio (OR) for dichromate traits or beta for quantitative traits according to PLINK [61].

Next, we evaluated the genetic relationships between these traits and schizophrenia using logistic regression. Only those traits with regression *p* values ≤0.15 in both the MGS and SSCCS and had the same direction of effect were included as potential classifiers for cluster analyses.

### 4.3. Cluster Analyses

We used the R platform to conduct our analyses. PRSs for schizophrenia [8] and the 10 selected traits were used in cluster analyses. The 10 selected traits were PGC Phase II bipolar disorder (BIP) [44]; body mass index (BMI) [62]; cannabis dependence (cannabis) [45]; ever smokers vs never smoker (evrSmk) [46]; working memory (MEM) [48]; verbal and numeric reasoning (VNR) [48]; neoopenness (OPEN) [49]; one person income per household (OPPH) [63]; subjective wellbeing (SWB) [47]; and years of schooling (YoS) [50]. We used the “NbClust” package [51] to explore the appropriate solution for the number of clusters based on Euclidean distances. The majority rule was used to select the number of clusters. In these analyses, the MGS dataset was used to explore likely cluster structure with different clustering parameters. Once a reasonable structure was found, the SSCCS dataset was used to validate the cluster structure using the same parameters. When a stable and consistent cluster structure was identified, the same parameters would be applied to the CATIE dataset to cluster the patients. The clusters from the CATIE dataset were used for membership association analyses with clinical, neurological, and laboratory data.

### 4.4. Clinical and Neurocognitive Data

The CATIE study was a double-blind randomized clinical trial to evaluate the effectiveness of antipsychotic drugs. There were 5 drugs used in the Phase I trial: perphenazine, olanzapine, quetiapine, risperidone, and ziprasidone. Participants were assigned a drug randomly and evaluated with extensive assessments for clinical symptoms, neurocognitive functions, and laboratory tests at the enrollment (baseline) and regular follow-ups for 18 months. We used the data obtained at the baseline, Visit 6 (about 6 months into Phase I), and at the end of the Phase I trial. Clinical symptoms were evaluated with the positive and negative syndrome scale (PANSS) [64]. Symptom count data were treated as quantitative measures without any transformation. We used the difference of symptom counts between the baseline and that at the end of Phase I trial to define treatment outcomes. If the difference was statistically significant, then the treatment outcome was judged to be effective. We used a paired *t*-test to compare the means for each symptom category separately. Data from a structured clinical interview for DSM IV (SCID), a clinical global impression (CGI), and treatment discontinuation were used in post hoc analyses. We used the neurocognitive data from the baseline and at Visit 6 to determine the improvement of neurocognitive functions. Neurocognitive functions, as defined in the CATIE study, included vigilance (VIGILANCE), processing speed (PROCESSING SPEED), reasoning (REASON), and working memory (WORKING MEMORY). We took the data as provided by NIMH Genetic Repository. More details of the CATIE neurocognitive tests were described elsewhere [65, 66].

### 4.5. Clinical Laboratory Test Data

The CATIE study had data for some standard laboratory tests at the enrollment and follow-up checks. We selected the data for bilirubin, uric acid, prolactin, and cell counts for neutrophils, eosinophils, lymphocytes, and monocytes for this study. The motivation for the inclusion of laboratory tests was to evaluate whether any of these tests had the potential for use as biomarkers for the classes resulting from our cluster analysis. The tests selected were related to oxidative stress, inflammation, hyperprolactinemia, and immune functions. In the literature, there were suggestions that oxidative stress could be an underlying factor for schizophrenia [67], and antipsychotic medicine led to increased levels of prolactin [68]. Dysregulation of the immune system and inflammation in patients of schizophrenia is well documented [69–71]. Both data at the baseline and at the end of Phase I were used.

### 4.6. Statistical Analyses

Once we grouped the patients into classes, we conducted analyses to evaluate whether these classes were associated with clinical features, treatment outcomes, neurocognitive functions, and laboratory tests by linear and logistic regression. Baseline assessments of clinical symptoms, neurocognitive and laboratory tests were used in these analyses where functional data were treated as quantitative outcomes and class memberships were treated as factorial predictors with Class I as a reference, sex, age, and assigned antipsychotics as covariates. The outcomes of Phase I treatment were evaluated by paired *t*-tests to compare the means of tested items between the baseline, Visit 6, and that at the end of Phase I trial. In all tests, *p* values were reported without multiple test correction.

## Figures and Tables

**Table 1:** Traits genetically related to schizophrenia.

Trait	MGS				SSCCS				CATIE			
	Estimate	Std.Err	*Z*	*Pr*(>∣*z*∣)	Estimate	Std.Err	*Z*	*Pr*(>∣*z*∣)	Estimate	Std.Err	*Z*	*Pr*(>∣*z*∣)
BMI	−317.20	198.30	−1.60	0.1096	−504.37	166.03	−3.04	0.0024	−876.75	384.79	−2.28	0.0227
Cannabis	30.67	19.17	1.60	0.1097	44.29	16.62	2.67	0.0077	33.10	37.81	0.88	0.3813
evrSmk	271.40	79.98	3.39	0.0007	354.79	69.68	5.09	3.55E-07	285.55	149.62	1.91	0.0563
MEM	1215.00	306.20	3.97	7.21E-05	1198.37	258.63	4.63	3.60E-06	837.03	563.61	1.49	0.1375
NEW_BIP	901.60	50.65	17.80	2.00E-16	582.88	41.94	13.90	2.00E-16	775.78	95.36	8.14	4.10E-16
Openness	36.04	14.06	2.56	0.0104	26.49	11.80	2.25	0.0247	74.71	26.85	2.78	0.0054
OPPH	−1215.00	606.10	−2.01	0.0449	−790.07	506.85	−1.56	0.1190	−759.07	1023.04	−0.74	0.4581
SWB	−716.70	312.00	−2.30	0.0216	−967.25	262.97	−3.68	0.0002	170.78	590.56	0.29	0.7724
VNR	−618.60	159.50	−3.88	0.0001	−455.89	138.07	−3.30	0.0010	−523.70	320.92	−1.63	0.1027
YoS	973.50	426.90	2.28	0.0226	1318.65	361.70	3.65	0.0003	−827.34	819.49	−1.01	0.3127

MGS: Molecular Genetics of Schizophrenia; SSCCS: Swedish Schizophrenia Case-Control Study; CATIE: Clinical Antipsychotic Trials for Intervention Effectiveness; BMI: body mass index; Cannabis: cannabis dependence; evrSmk: ever smoker vs never smoker; MEM: working memory; NEW_BIP: PGC Phase II bipolar disorder; Openness: neoopenness; OPPH: one person income per household; SWB: subjective wellbeing; VNR: verbal and numeric reasoning; YoS: years of school attended.

**Table 2:** Symptom count changes between the baseline and the end of Phase I trial.

	All subjects	Class I	Class II	Class III	Class IV	Class V
*Total symptom*						
Mean Diff	4.17	5.47	3.90	1.12	4.89	3.41
95% CI	2.59-5.75	2.76-8.19	0.78-7.03	−3.34-5.58	−0.12-9.90	−0.32-7.14
*t*	5.17	3.98	2.48	3.18	1.97	1.82
*df*	435	171	81	56	44	79
*P*	**3.50E-07**	**1.02E-04**	**0.0151**	0.6160	0.0557	0.0725

*Positive symptom*						
Mean Diff	1.18	1.26	1.02	0.82	1.60	1.20
95% CI	0.63-1.73	0.31-2.22	0.00-2.05	−0.69-2.34	−0.22-3.42	−0.10-2.50
*t*	4.24	2.61	2.00	1.09	1.77	1.83
*df*	435	171	81	56	44	79
*P*	**2.78E-05**	**9.90E-03**	**0.0492**	0.2801	0.0839	0.0707

*Negative symptom*						
Mean Diff	0.93	1.36	0.65	−0.63	2.18	0.73
95% CI	0.39-1.48	0.47-2.25	−0.54-1.83	−2.32-1.05	0.53-3.82	−0.49-1.94
*t*	3.38	3.03	1.08	−0.75	2.65	1.19
*df*	435	171	81	56	44	79
*P*	**7.94E-04**	**2.81E-03**	0.2817	0.4556	**0.0111**	0.2382

Mean Diff: mean difference between the baseline and at the end of Phase I trial; 95% CI: 95% confidence interval; t: test *t* value; df: degree of freedom; *P* values ≤0.05 were highlighted by bold.

**Table 3:** Change of cognitive functions between baseline and Visit 6.

	Mean Diff	*t*-value	df	95% CI	*p* value
*VIGILANCE*					
**All subjects**	**−0.30**	**−5.31**	**200**	**−0.40–−0.19**	**3.0E-07**
**Class I**	**−0.23**	**−2.64**	**79**	**−0.40–−0.06**	**0.0099**
Class II	−0.24	−1.45	33	−0.59–0.10	0.1578
**Class III**	**−0.41**	**−2.97**	**24**	**−0.70–−0.13**	**0.0066**
**Class IV**	**−0.34**	**−2.41**	**23**	**−0.63–−0.05**	**0.0242**
**Class V**	**−0.38**	**−3.03**	**37**	**−0.63–−0.13**	**0.0045**

*PROCESSING SPEED*					
**All subjects**	**−0.24**	**−6.45**	**224**	**−0.32–−0.17**	**6.8E-10**
**Class I**	**−0.20**	**−3.56**	**87**	**−0.31–−0.09**	**0.0006**
**Class II**	**−0.29**	**−2.85**	**38**	**−0.50–−0.08**	**0.0071**
**Class III**	**−0.29**	**−2.41**	**27**	**−0.54–−0.04**	**0.0229**
**Class IV**	**−0.26**	**−2.17**	**26**	**−0.51–−0.01**	**0.0395**
**Class V**	**−0.25**	**−3.23**	**42**	**−0.40–−0.09**	**0.0024**

*WORKING MEMORY*					
**All subjects**	**−0.20**	**−4.27**	**224**	**−0.29–−0.11**	**2.8E-05**
**Class I**	**−0.21**	**−3.14**	**87**	**−0.34–−0.08**	**0.0023**
Class II	−0.18	−1.36	38	−0.44–0.09	0.1819
Class III	−0.09	−0.57	27	−0.44–0.25	0.5740
**Class IV**	**−0.26**	**−2.12**	**26**	**−0.52–−0.01**	**0.0433**
**Class V**	**−0.21**	**−2.32**	**42**	**−0.39–−0.03**	**0.0252**

*REASONING*					
**All subjects**	**−0.27**	**−5.15**	**224**	**−0.38–−0.17**	**5.8E-07**
**Class I**	**−0.28**	**−3.27**	**87**	**−0.45–−0.11**	**0.0015**
Class II	−0.24	−1.95	38	−0.50–0.01	0.0585
Class III	−0.27	−1.67	27	−0.60–0.06	0.1056
**Class IV**	**−0.40**	**−2.78**	**26**	**−0.69–−0.10**	**0.0100**
Class V	−0.20	−1.66	42	−0.44–0.04	0.1053

Mean Diff: mean difference between the baseline and Visit 6; 95% CI: 95% confident interval; *t*-value: Test *t* value; df: Degree of freedom; *P* values ≤0.05 were highlighted in bold.

**Table 4:** Changes of laboratory tests between baseline and end of Phase I trial.

	Mean Diff	*t*-value	df	95% CI	*p* value
*HDL*					
All subjects	−0.07	−0.15	419	−0.99–0.85	0.8833
**Class I**	**−2.25**	**−3.04**	**168**	**−3.70–−0.79**	**0.0027**
Class II	−0.68	−0.61	78	−2.90–1.53	0.5406
Class III	2.94	1.98	49	−0.05–5.93	0.0537
Class IV	1.93	1.55	43	−0.57–4.44	0.1274
**Class V**	**2.22**	**2.30**	**77**	**0.30–4.13**	**0.0239**

*Calcium*					
**All subjects**	**0.08**	**3.61**	**419**	**0.04–0.12**	**0.0003**
Class I	0.05	1.28	168	−0.02–0.13	0.2041
Class II	0.08	1.80	77	−0.01–0.18	0.0763
Class III	0.07	1.10	50	−0.06–0.21	0.2757
Class IV	0.12	1.79	43	−0.02–0.26	0.0806
**Class V**	**0.14**	**2.71**	**77**	**0.04–0.24**	**0.0084**

*Phosphorus*					
**All subjects**	**0.10**	**2.99**	**419**	**0.04–0.17**	**0.0029**
Class I	0.02	0.41	168	−0.09–0.14	0.6858
Class II	0.12	1.49	77	−0.04–0.28	0.1409
**Class III**	**0.19**	**2.26**	**50**	**0.02–0.37**	**0.0282**
Class IV	0.16	1.54	43	−0.05–0.38	0.1318
**Class V**	**0.17**	**2.25**	**77**	**0.02–0.32**	**0.0276**

*Lymphocytes*					
All subjects	0.23	0.55	416	−0.58–1.04	0.5806
Class I	0.67	1.06	164	−0.58–1.90	0.2915
Class II	0.68	0.68	78	−1.11–2.47	0.4531
Class III	−0.14	−0.12	52	−2.53–2.25	0.9084
**Class IV**	**−3.12**	**−2.86**	**43**	**−5.32–−0.92**	**0.0065**
Class V	1.00	0.90	75	−1.21–3.21	0.3684

*Neutrophils*					
All subjects	−0.48	−1.02	416	−1.40–0.44	0.3092
Class I	−0.96	−1.42	164	−2.30–0.38	0.1588
Class II	−0.98	−0.91	78	−3.12–1.16	0.3660
Class III	0.34	0.23	52	−2.58–3.26	0.8161
**Class IV**	**3.04**	**2.39**	**43**	**0.47–5.61**	**0.0214**
Class V	−1.51	−1.22	75	−3.97–0.96	0.2265

Mean Diff: mean difference between the baseline and Phase I; 95% CI: 95% confidence interval; *t*-value: test *t* value; df: degree of freedom; *P* values ≤0.05 were highlighted by bold; HDL: high-density lipoproteins.

**Table 5:** A summary of subtypes classified by partially shared liability between schizophrenia and comorbid traits.

Class	# subjects (%)	Symptom improvement	Cognitive function	Laboratory test	Other clinical features
Class I	181 (39.3%)	Total symptom ↓ Positive ↓ Negative ↓	VIGILANCE ↑ PROCESSING SPEED ↑ MEMORY ↑ REASONING ↑	HDL ↓	Reference

Class II	94 (18.6%)	Total symptom ↓ Positive ↓	PROCESSING SPEED ↑	Bilirubin ↓ Uric acid ↓	Lower baseline negative symptoms, lower bilirubin and uric acid, more likely to use tobacco product

Class III	64 (12.9%)	—	VIGILANCE ↑	Phosphorus ↑	

Class IV	53 (10.1%)	Negative ↓	VIGILANCE ↑ PROCESSING SPEED ↑ MEMORY ↑ REASONING ↑	Lymphocyte ↓ Neutrophils ↑	More likely to have a family history and an older age when first prescribed antipsychotic medicine, less likely to discontinue medication due to the lack of effects

Class V	90 (18.1%)	—	VIGILANCE ↑ PROCESSING SPEED ↑	HDL ↑ Calcium ↑ Phosphorus ↑ Prolactin ↑	

## Data Availability

All data used in this study were obtained from the NIMH Genetic Repository (https://www.nimhgenetics.org/) and are available to qualified researchers.

## Supplementary Materials

Supplementary Materials Supplementary Table S1: traits screened for genetic relationship with schizophrenia. Table S2: baseline laboratory test in Classes II to V as compared to Class I. Table S3: class association with age first prescribed antipsychotics and family history. Table S4: class association with the use of tobacco products. Table S5: class association with treatment discontinuation. Table S6: Phase I discontinuation outcome.

## References

[1] Albus M. (2012). Clinical courses of schizophrenia. *Pharmacopsychiatry*.

[2] Heilbronner U., Samara M., Leucht S., Falkai P., Schulze T. G. (2016). The longitudinal course of schizophrenia across the lifespan: clinical, cognitive, and neurobiological aspects. *Harvard Review of Psychiatry*.

[3] Tandon R., Nasrallah H. A., Keshavan M. S. (2009). Schizophrenia, “just the facts” 4. Clinical features and conceptualization. *Schizophrenia Research*.

[4] Jablensky A. (2010). The diagnostic concept of schizophrenia: its history, evolution, and future prospects. *Dialogues in Clinical Neuroscience*.

[5] Sullivan P. F., Kendler K. S., Neale M. C. (2003). Schizophrenia as a complex trait: evidence from a meta-analysis of twin studies. *Archives of General Psychiatry*.

[6] The Schizophrenia Psychiatric Genome-Wide Association Study (GWAS) Consortium (2011). Genome-wide association study identifies five new schizophrenia loci. *Nature Genetics*.

[7] Ripke S., O'Dushlaine C., Chambert K. (2013). Genome-wide association analysis identifies 13 new risk loci for schizophrenia. *Nature Genetics*.

[8] Schizophrenia Working Group of the Psychiatric Genomics Consortium (2014). Biological insights from 108 schizophrenia-associated genetic loci. *Nature*.

[9] Li Z., Chen J., Yu H. (2017). Genome-wide association analysis identifies 30 new susceptibility loci for schizophrenia. *Nature Genetics*.

[10] Edwards A. C., Bigdeli T. B., Docherty A. R. (2016). Meta-analysis of positive and negative symptoms reveals schizophrenia modifier genes. *Schizophrenia Bulletin*.

[11] Kay S. R. (1990). Positive-negative symptom assessment in schizophrenia: psychometric issues and scale comparison. *The Psychiatric Quarterly*.

[12] Goldstein G. (1994). Neurobehavioral heterogeneity in schizophrenia. *Archives of Clinical Neuropsychology*.

[13] Cadoo K. A., Traina T. A., King T. A. (2013). Advances in molecular and clinical subtyping of breast cancer and their implications for therapy. *Surgical Oncology Clinics of North America*.

[14] Dawson S.-J., Rueda O. M., Aparicio S., Caldas C. (2013). A new genome-driven integrated classification of breast cancer and its implications. *The EMBO Journal*.

[15] Bogiatzi C., Wannarong T., McLeod A. I., Heisel M., Hackam D., Spence J. D. (2014). SPARKLE (Subtypes of Ischaemic Stroke Classification System), incorporating measurement of carotid plaque burden: a new validated tool for the classification of ischemic stroke subtypes. *Neuroepidemiology*.

[16] Rannikmäe K., Woodfield R., Anderson C. S. (2016). Reliability of intracerebral hemorrhage classification systems: a systematic review. *International Journal of Stroke*.

[17] Fenton W. S., McGlashan T. H. (1991). Natural history of schizophrenia Subtypes. *Archives of General Psychiatry*.

[18] Dollfus S., Everitt B., Ribeyre J. M., Assouly-Besse F., Sharp C., Petit M. (1996). Identifying subtypes of schizophrenia by cluster analyses. *Schizophrenia Bulletin*.

[19] McGlashan T. H., Fenton W. S. (1991). Classical subtypes for schizophrenia: literature review for DSM-IV. *Schizophrenia Bulletin*.

[20] Roy M. A., Mérette C., Maziade M. (2001). Subtyping schizophrenia according to outcome or severity: a search for homogeneous subgroups. *Schizophrenia Bulletin*.

[21] Brazo P., Marié R. M., Halbecq I. (2002). Cognitive patterns in subtypes of schizophrenia. *European Psychiatry*.

[22] Fervaha G., Agid O., Foussias G., Siddiqui I., Takeuchi H., Remington G. (2016). Neurocognitive impairment in the deficit subtype of schizophrenia. *European Archives of Psychiatry and Clinical Neuroscience*.

[23] Gould I. C., Shepherd A. M., Laurens K. R., Cairns M. J., Carr V. J., Green M. J. (2014). Multivariate neuroanatomical classification of cognitive subtypes in schizophrenia: a support vector machine learning approach. *NeuroImage: Clinica*.

[24] Hallmayer J. F., Kalaydjieva L., Badcock J. (2005). Genetic evidence for a distinct subtype of schizophrenia characterized by pervasive cognitive deficit. *American Journal of Human Genetics*.

[25] Rocca P., Galderisi S., Rossi A. (2016). Social cognition in people with schizophrenia: a cluster-analytic approach. *Psychological Medicine*.

[26] Voineskos A. N., Foussias G., Lerch J. (2013). Neuroimaging evidence for the deficit subtype of schizophrenia. *JAMA Psychiatry*.

[27] Beratis S., Gabriel J., Hoidas S. (1994). Age at onset in subtypes of schizophrenic disorders. *Schizophrenia Bulletin*.

[28] Maglione J. E., Thomas S. E., Jeste D. V. (2014). Late-onset schizophrenia: do recent studies support categorizing LOS as a subtype of schizophrenia?. *Current Opinion in Psychiatry*.

[29] Butcher N. J., Fung W. L. A., Fitzpatrick L. (2015). Response to clozapine in a clinically identifiable subtype of schizophrenia. *The British Journal of Psychiatry: the Journal of Mental Science*.

[30] Farooq S., Agid O., Foussias G., Remington G. (2013). Using treatment response to subtype schizophrenia: proposal for a new paradigm in classification. *Schizophrenia Bulletin*.

[31] Gillespie A. L., Samanaite R., Mill J., Egerton A., MacCabe J. H. (2017). Is treatment-resistant schizophrenia categorically distinct from treatment-responsive schizophrenia? A systematic review. *BMC Psychiatry*.

[32] Bassett A. S., Chow E. W. C. (1999). 22q11 deletion syndrome: a genetic subtype of schizophrenia. *Biological Psychiatry*.

[33] Green M. J., on behalf of the Australian Schizophrenia Research Bank, Cairns M. J. (2013). Genome-wide supported variant MIR137 and severe negative symptoms predict membership of an impaired cognitive subtype of schizophrenia. *Molecular Psychiatry*.

[34] Holliday E. G., McLean D. E., Nyholt D. R., Mowry B. J. (2009). Susceptibility locus on chromosome 1q23-25 for a schizophrenia subtype resembling deficit schizophrenia identified by latent class analysis. *Archives of General Psychiatry*.

[35] Wessman J., Paunio T., Tuulio-Henriksson A. (2009). Mixture Model Clustering of Phenotype Features Reveals Evidence for Association of DTNBP1 to a Specific Subtype of Schizophrenia. *Biological Psychiatry*.

[36] Welter D., MacArthur J., Morales J. (2013). The NHGRI GWAS Catalog, a curated resource of SNP-trait associations. *Nucleic Acids Research*.

[37] Sivakumaran S., Agakov F., Theodoratou E. (2011). Abundant pleiotropy in human complex diseases and traits. *American Journal of Human Genetics*.

[38] Solovieff N., Cotsapas C., Lee P. H., Purcell S. M., Smoller J. W. (2013). Pleiotropy in complex traits: challenges and strategies. *Nature Reviews. Genetics*.

[39] Bulik-Sullivan B., Consortium R. G., Finucane H. K. (2015). An atlas of genetic correlations across human diseases and traits. *Nature Genetics*.

[40] Westermeyer J. (2006). Comorbid schizophrenia and substance abuse: a review of epidemiology and course. *The American Journal on Addictions*.

[41] Buckley P. F., Miller B. J., Lehrer D. S., Castle D. J. (2009). Psychiatric Comorbidities and Schizophrenia. *Schizophrenia Bulletin*.

[42] Jeste D. V., Gladsjo J. A., Lindamer L. A., Lacro J. P. (1996). Medical comorbidity in schizophrenia. *Schizophrenia Bulletin*.

[43] Shi J., Levinson D. F., Duan J. (2009). Common variants on chromosome 6p22.1 are associated with schizophrenia. *Nature*.

[44] Hou L., Bergen S. E., Akula N. (2016). Genome-wide association study of 40,000 individuals identifies two novel loci associated with bipolar disorder. *Human Molecular Genetics*.

[45] Sherva R., Wang Q., Kranzler H. (2016). Genome-wide Association Study of Cannabis Dependence Severity, Novel Risk Variants, and Shared Genetic Risks. *JAMA Psychiatry*.

[46] (2010). Genome-wide meta-analyses identify multiple loci associated with smoking behavior. *Nature Genetics*.

[47] Harris S. E., Hagenaars S. P., Davies G. (2017). Molecular genetic contributions to self-rated health. *International Journal of Epidemiology*.

[48] Davies G., Marioni R. E., Liewald D. C. (2016). Genome-wide association study of cognitive functions and educational attainment in UK Biobank (N=112 151). *Molecular Psychiatry*.

[49] de Moor M. H. M., Costa P. T., Terracciano A. (2012). Meta-analysis of genome-wide association studies for personality. *Molecular Psychiatry*.

[50] Okbay A., Beauchamp J. P., Fontana M. A. (2016). Genome-wide association study identifies 74 loci associated with educational attainment. *Nature*.

[51] Charrad M., Ghazzali N., Boiteau V., Niknafs A. (2014). NbClust: AnRPackage for Determining the Relevant Number of Clusters in a Data Set. *Journal of Statistical Software*.

[52] Stroup T. S., McEvoy J. P., Swartz M. S. (2003). The National Institute of Mental Health Clinical Antipsychotic Trials of Intervention Effectiveness (CATIE) project: schizophrenia trial design and protocol development. *Schizophrenia Bulletin*.

[53] Sullivan P. F., Lin D., Tzeng J. Y. (2008). Genomewide association for schizophrenia in the CATIE study: results of stage 1. *Molecular Psychiatry*.

[54] Chen X., Li Y., Zhang T., Yao Y., Shen C., Xue Y. (2018). Association of serum trace elements with schizophrenia and effects of antipsychotic treatment. *Biological Trace Element Research*.

[55] Zahorec R. (2001). Ratio of neutrophil to lymphocyte counts--rapid and simple parameter of systemic inflammation and stress in critically ill. *Bratislavské Lekárske Listy*.

[56] Özdin S., Böke Ö. (2019). Neutrophil/lymphocyte, platelet/lymphocyte and monocyte/lymphocyte ratios in different stages of schizophrenia. *Psychiatry Research*.

[57] Nitta M., Kishimoto T., Müller N. (2013). Adjunctive use of nonsteroidal anti-inflammatory drugs for schizophrenia: a meta-analytic investigation of randomized controlled trials. *Schizophrenia Bulletin*.

[58] Howie B., Fuchsberger C., Stephens M., Marchini J., Abecasis G. R. (2012). Fast and accurate genotype imputation in genome-wide association studies through pre-phasing. *Nature Genetics*.

[59] Ware J. J., Chen X., Vink J. (2016). Genome-Wide Meta-Analysis of Cotinine Levels in Cigarette Smokers Identifies Locus at 4q13.2. *Scientific Reports*.

[60] The International Schizophrenia Consortium (2009). Common polygenic variation contributes to risk of schizophrenia and bipolar disorder. *Nature*.

[61] Purcell S., Neale B., Todd-Brown K. (2007). PLINK: a tool set for whole-genome association and population-based linkage analyses. *American Journal of Human Genetics*.

[62] Graff M., Scott R. A., Justice A. E. (2017). Genome-wide physical activity interactions in adiposity - a meta-analysis of 200,452 adults. *PLoS Genetics*.

[63] Hill W. D., Hagenaars S. P., Marioni R. E. (2016). Molecular genetic contributions to social deprivation and household income in UK biobank. *Current Biology*.

[64] Kay S. R., Fiszbein A., Opler L. A. (1987). The positive and negative syndrome scale (PANSS) for schizophrenia. *Schizophrenia Bulletin*.

[65] Keefe R. S. E., Mohs R. C., Bilder R. M. (2003). Neurocognitive assessment in the Clinical Antipsychotic Trials of Intervention Effectiveness (CATIE) project schizophrenia trial: development, methodology, and rationale. *Schizophrenia Bulletin*.

[66] Keefe R. S. E., Bilder R. M., Harvey P. D. (2046). Baseline Neurocognitive Deficits in the CATIE Schizophrenia Trial. *Neuropsychopharmacology*.

[67] Flatow J., Buckley P., Miller B. J. (2013). Meta-analysis of oxidative stress in schizophrenia. *Biological Psychiatry*.

[68] Leucht S., Cipriani A., Spineli L. (2013). Comparative efficacy and tolerability of 15 antipsychotic drugs in schizophrenia: a multiple-treatments meta-analysis. *The Lancet*.

[69] Bergink V., Gibney S. M., Drexhage H. A. (2014). Autoimmunity, inflammation, and psychosis: a search for peripheral markers. *Biological Psychiatry*.

[70] Meyer U. (2013). Developmental neuroinflammation and schizophrenia. *Progress in Neuro-Psychopharmacology and Biological Psychiatry*.

[71] Müller N., Weidinger E., Leitner B., Schwarz M. J. (2015). The role of inflammation in schizophrenia. *Frontiers in Neuroscience*.

