# Supplementary material for: Polygenic Risk Scores for Subtyping of Schizophrenia
Source: Schizophr Res Treatment. 2020 Jul 23;2020:1638403. doi: 10.1155/2020/1638403 (PMC7396092; doi:10.1155/2020/1638403)
Supplement: Supplementary Materials — Supplementary Table S1: traits screened for genetic relationship with schizophrenia. Table S2: baseline laboratory test in Classes II to V as compared to Class I. Table S3: class association with age first prescribed antipsychotics and family history. Table S4: class association with the use of tobacco products. Table S5: class association with treatment discontinuation. Table S6: Phase I discontinuation outcome. [file 1638403.f1.docx]

Table S1. Traits screened for genetic relationship with schizophrenia

| **Traits** | **Abbre-viation** | **Source** | **Reference** |
| --- | --- | --- | --- |
| Anorexia | ANO | http://www.med.unc.edu/pgc/files/resultfiles/gcan_meta-out.gz/view | (Boraska et al., 2014) |
| Anxiety spectrum disorder | ASD | https://www.med.unc.edu/pgc/results-and-downloads |  |
| Bipolar disorder | BIP | https://www.med.unc.edu/pgc/results-and-downloads | (Psychiatric GWAS Consortium Bipolar Disorder Working Group, 2011, p. 4) |
| Bipolar disorder II | BIP_II | https://www.nimh.nih.gov/labs-at-nimh/research-areas/clinics-and-labs/hgb/data-downloads.shtml | (Hou et al., 2016) |
| Body mass index | BMI | http://portals.broadinstitute.org/collaboration/giant/index.php/GIANT_consortium_data_files | (Graff et al., 2017) |
| Coronary artery disease | CAD | http://www.cardiogramplusc4d.org/data-downloads/ | (Nikpay et al., 2015) |
| Cannabis dependence | CAN | From the authors of the publication | (Sherva et al., 2016) |
| Crohn’s disease | CD | https://www.ibdgenetics.org/downloads.html | (Barrett et al., 2008) |
| Cigarette smoked  Per day | CPD | https://www.med.unc.edu/pgc/results-and-downloads | (Tobacco and Genetics Consortium, 2010) |
| Depressive symptoms | DS | https://www.ebi.ac.uk/gwas/downloads/summary-statistics | (Okbay et al., 2016a) |
| Early vs late person | earlyLate | https://www.ebi.ac.uk/gwas/downloads/summary-statistics | (Hu et al., 2016) |
| Ever smokers | evrSmk | https://www.med.unc.edu/pgc/results-and-downloads | (Tobacco and Genetics Consortium, 2010) |
| Former smokers | fmrSmk | https://www.med.unc.edu/pgc/results-and-downloads | (Tobacco and Genetics Consortium, 2010) |
| Inflammatory Bowel disease | IBD | https://www.ibdgenetics.org/downloads.html | (Liu et al., 2015) |
| Household income | Income | http://www.ccace.ed.ac.uk/node/335 | (Hill et al., 2016) |
| Internalization | INT | https://www.med.unc.edu/pgc/results-and-downloads | (Benke et al., 2014) |
| Major depressive disorder | MDD | https://www.med.unc.edu/pgc/results-and-downloads | (Major Depressive Disorder Working Group of the Psychiatric GWAS Consortium et al., 2013) |
| Working memory | MEM | https://www.ebi.ac.uk/gwas/downloads/summary-statistics | (Davies et al., 2016) |
| Neuroticism | NEU | https://www.ebi.ac.uk/gwas/downloads/summary-statistics | (Okbay et al., 2016a) |
| Neo-openness | Open | http://www.tweelingenregister.org/GPC/ | (de Moor et al., 2012) |
| One person income per household | OPPH | http://www.ccace.ed.ac.uk/node/335 | (Hill et al., 2016) |
| Smoking age onset | SmkAgeonset | https://www.med.unc.edu/pgc/results-and-downloads | (Tobacco and Genetics Consortium, 2010) |
| Subjective well being | SWB | https://www.ebi.ac.uk/gwas/downloads/summary-statistics | (Harris et al., 2016) |
| Verbal and numeric reasoning | VNR | https://www.ebi.ac.uk/gwas/downloads/summary-statistics | (Davies et al., 2011) |
| Years of schooling | YoS | https://www.thessgac.org/data | (Okbay et al., 2016b) |

**Table S2. Baseline laboratory test results**

|  | **Estimate** | **Std. Error** | **t value** | **Pr(>\|t\|)** |
| --- | --- | --- | --- | --- |
| **Bilirubin** | | | | |
| **Class II** | **-0.09** | **0.03** | **-2.77** | **0.0058** |
| Class III | -0.05 | 0.04 | -1.21 | 0.2282 |
| Class IV | -0.07 | 0.04 | -1.63 | 0.1036 |
| Class V | -0.06 | 0.03 | -1.89 | 0.0595 |
| **Prolactin** | | | | |
| Class II | 4.31 | 4.55 | 0.95 | 0.3434 |
| Class III | -1.07 | 5.17 | -0.21 | 0.8361 |
| Class IV | 1.16 | 5.64 | 0.21 | 0.8371 |
| **Class V** | **9.74** | **4.65** | **2.10** | **0.0365** |
| **Uric Acid** | | | | |
| **Class II** | **-0.46** | **0.21** | **-2.26** | **0.0243** |
| Class III | -0.12 | 0.24 | -0.52 | 0.6064 |
| Class IV | -0.41 | 0.25 | -1.66 | 0.0986 |
| Class V | 0.13 | 0.21 | 0.64 | 0.5238 |

**Table S3. Class association with treatment discontinuation**

|  | **Estimate** | **Std. Error** | **z value** | **Pr(>\|z\|)** |
| --- | --- | --- | --- | --- |
| **Likelihood to Discontinue Due to Lack of Effect** | | | | |
| Class II | -0.18 | 0.28 | -0.65 | 0.5174 |
| Class III | 0.02 | 0.31 | 0.05 | 0.9584 |
| **Class IV** | **-0.87** | **0.40** | **-2.17** | **0.0299** |
| Class V | -0.21 | 0.28 | -0.74 | 0.4617 |

**Table S4. Class association with age first prescribed antipsychotics and family history**

|  | **Estimate** | **Std. Error** | **t value** | **Pr(>\|t\|)** |
| --- | --- | --- | --- | --- |
| **Age at Antipsychotics** | | | | |
| Class II | 0.54 | 1.04 | 0.52 | 0.6047 |
| Class III | -0.80 | 1.21 | -0.66 | 0.5083 |
| **Class IV** | **2.70** | **1.28** | **2.10** | **0.0363** |
| Class V | -0.06 | 1.06 | -0.06 | 0.9538 |
| **Family History of Mental Illness** | | | | |
| Class II | -0.05 | 0.26 | -0.18 | 0.8606 |
| Class III | 0.14 | 0.30 | 0.48 | 0.6319 |
| **Class IV** | **0.69** | **0.32** | **2.13** | **0.0331** |
| Class V | 0.28 | 0.26 | 1.08 | 0.2803 |

**Table S5. Class association with the use of tobacco products**

|  | **Estimate** | **Std. Error** | **t value** | **Pr(>\|t\|)** |
| --- | --- | --- | --- | --- |
| **Tobacco Use** | | | | |
| **Class II** | **0.64** | **0.28** | **2.29** | **0.0219** |
| Class III | 0.07 | 0.30 | 0.23 | 0.8222 |
| Class IV | 0.61 | 0.35 | 1.76 | 0.0786 |
| Class V | 0.10 | 0.26 | 0.37 | 0.7115 |

**Table S6. Phase I discontinuation outcome**

|  | **Estimate** | **Std. Error** | **z value** | **Pr(>\|z\|)** |
| --- | --- | --- | --- | --- |
| **Likelihood to Discontinue Due to Lack of Effect** | | | | |
| Class II | -0.18 | 0.28 | -0.65 | 0.5174 |
| Class III | 0.02 | 0.31 | 0.05 | 0.9584 |
| **Class IV** | **-0.87** | **0.40** | **-2.17** | **0.0299** |
| Class V | -0.21 | 0.28 | -0.74 | 0.4617 |

References

Barrett, J.C., Hansoul, S., Nicolae, D.L., Cho, J.H., Duerr, R.H., Rioux, J.D., Brant, S.R., Silverberg, M.S., Taylor, K.D., Barmada, M.M., Bitton, A., Dassopoulos, T., Datta, L.W., Green, T., Griffiths, A.M., Kistner, E.O., Murtha, M.T., Regueiro, M.D., Rotter, J.I., Schumm, L.P., Steinhart, A.H., Targan, S.R., Xavier, R.J., NIDDK IBD Genetics Consortium, Libioulle, C., Sandor, C., Lathrop, M., Belaiche, J., Dewit, O., Gut, I., Heath, S., Laukens, D., Mni, M., Rutgeerts, P., Van Gossum, A., Zelenika, D., Franchimont, D., Hugot, J.-P., de Vos, M., Vermeire, S., Louis, E., Belgian-French IBD Consortium, Wellcome Trust Case Control Consortium, Cardon, L.R., Anderson, C.A., Drummond, H., Nimmo, E., Ahmad, T., Prescott, N.J., Onnie, C.M., Fisher, S.A., Marchini, J., Ghori, J., Bumpstead, S., Gwilliam, R., Tremelling, M., Deloukas, P., Mansfield, J., Jewell, D., Satsangi, J., Mathew, C.G., Parkes, M., Georges, M., Daly, M.J., 2008. Genome-wide association defines more than 30 distinct susceptibility loci for Crohn’s disease. Nat. Genet. 40, 955–962. https://doi.org/10.1038/ng.175

Benke, K.S., Nivard, M.G., Velders, F.P., Walters, R.K., Pappa, I., Scheet, P.A., Xiao, X., Ehli, E.A., Palmer, L.J., Whitehouse, A.J.O., Verhulst, F.C., Jaddoe, V.W., Rivadeneira, F., Groen-Blokhuis, M.M., van Beijsterveldt, C.E.M., Davies, G.E., Hudziak, J.J., Lubke, G.H., Boomsma, D.I., Pennell, C.E., Tiemeier, H., Middeldorp, C.M., 2014. A Genome-wide Association Meta-analysis of Preschool Internalizing Problems. J. Am. Acad. Child Adolesc. Psychiatry 53, 667-676.e7. https://doi.org/10.1016/j.jaac.2013.12.028

Boraska, V., Franklin, C.S., Floyd, J. a. B., Thornton, L.M., Huckins, L.M., Southam, L., Rayner, N.W., Tachmazidou, I., Klump, K.L., Treasure, J., Lewis, C.M., Schmidt, U., Tozzi, F., Kiezebrink, K., Hebebrand, J., Gorwood, P., Adan, R. a. H., Kas, M.J.H., Favaro, A., Santonastaso, P., Fernández-Aranda, F., Gratacos, M., Rybakowski, F., Dmitrzak-Weglarz, M., Kaprio, J., Keski-Rahkonen, A., Raevuori, A., Van Furth, E.F., Slof-Op ’t Landt, M.C.T., Hudson, J.I., Reichborn-Kjennerud, T., Knudsen, G.P.S., Monteleone, P., Kaplan, A.S., Karwautz, A., Hakonarson, H., Berrettini, W.H., Guo, Y., Li, D., Schork, N.J., Komaki, G., Ando, T., Inoko, H., Esko, T., Fischer, K., Männik, K., Metspalu, A., Baker, J.H., Cone, R.D., Dackor, J., DeSocio, J.E., Hilliard, C.E., O’Toole, J.K., Pantel, J., Szatkiewicz, J.P., Taico, C., Zerwas, S., Trace, S.E., Davis, O.S.P., Helder, S., Bühren, K., Burghardt, R., de Zwaan, M., Egberts, K., Ehrlich, S., Herpertz-Dahlmann, B., Herzog, W., Imgart, H., Scherag, A., Scherag, S., Zipfel, S., Boni, C., Ramoz, N., Versini, A., Brandys, M.K., Danner, U.N., de Kovel, C., Hendriks, J., Koeleman, B.P.C., Ophoff, R.A., Strengman, E., van Elburg, A.A., Bruson, A., Clementi, M., Degortes, D., Forzan, M., Tenconi, E., Docampo, E., Escaramís, G., Jiménez-Murcia, S., Lissowska, J., Rajewski, A., Szeszenia-Dabrowska, N., Slopien, A., Hauser, J., Karhunen, L., Meulenbelt, I., Slagboom, P.E., Tortorella, A., Maj, M., Dedoussis, G., Dikeos, D., Gonidakis, F., Tziouvas, K., Tsitsika, A., Papezova, H., Slachtova, L., Martaskova, D., Kennedy, J.L., Levitan, R.D., Yilmaz, Z., Huemer, J., Koubek, D., Merl, E., Wagner, G., Lichtenstein, P., Breen, G., Cohen-Woods, S., Farmer, A., McGuffin, P., Cichon, S., Giegling, I., Herms, S., Rujescu, D., Schreiber, S., Wichmann, H.-E., Dina, C., Sladek, R., Gambaro, G., Soranzo, N., Julia, A., Marsal, S., Rabionet, R., Gaborieau, V., Dick, D.M., Palotie, A., Ripatti, S., Widén, E., Andreassen, O.A., Espeseth, T., Lundervold, A., Reinvang, I., Steen, V.M., Le Hellard, S., Mattingsdal, M., Ntalla, I., Bencko, V., Foretova, L., Janout, V., Navratilova, M., Gallinger, S., Pinto, D., Scherer, S.W., Aschauer, H., Carlberg, L., Schosser, A., Alfredsson, L., Ding, B., Klareskog, L., Padyukov, L., Courtet, P., Guillaume, S., Jaussent, I., Finan, C., Kalsi, G., Roberts, M., Logan, D.W., Peltonen, L., Ritchie, G.R.S., Barrett, J.C., Wellcome Trust Case Control Consortium 3, Estivill, X., Hinney, A., Sullivan, P.F., Collier, D.A., Zeggini, E., Bulik, C.M., 2014. A genome-wide association study of anorexia nervosa. Mol. Psychiatry 19, 1085–1094. https://doi.org/10.1038/mp.2013.187

Davies, G., Marioni, R.E., Liewald, D.C., Hill, W.D., Hagenaars, S.P., Harris, S.E., Ritchie, S.J., Luciano, M., Fawns-Ritchie, C., Lyall, D., Cullen, B., Cox, S.R., Hayward, C., Porteous, D.J., Evans, J., McIntosh, A.M., Gallacher, J., Craddock, N., Pell, J.P., Smith, D.J., Gale, C.R., Deary, I.J., 2016. Genome-wide association study of cognitive functions and educational attainment in UK Biobank (N=112 151). Mol. Psychiatry 21, 758–767. https://doi.org/10.1038/mp.2016.45

Davies, G., Tenesa, A., Payton, A., Yang, J., Harris, S.E., Liewald, D., Ke, X., Le Hellard, S., Christoforou, A., Luciano, M., McGhee, K., Lopez, L., Gow, A.J., Corley, J., Redmond, P., Fox, H.C., Haggarty, P., Whalley, L.J., McNeill, G., Goddard, M.E., Espeseth, T., Lundervold, A.J., Reinvang, I., Pickles, A., Steen, V.M., Ollier, W., Porteous, D.J., Horan, M., Starr, J.M., Pendleton, N., Visscher, P.M., Deary, I.J., 2011. Genome-wide association studies establish that human intelligence is highly heritable and polygenic. Mol. Psychiatry 16, 996–1005. https://doi.org/10.1038/mp.2011.85

de Moor, M.H.M., Costa, P.T., Terracciano, A., Krueger, R.F., de Geus, E.J.C., Toshiko, T., Penninx, B.W.J.H., Esko, T., Madden, P. a. F., Derringer, J., Amin, N., Willemsen, G., Hottenga, J.-J., Distel, M.A., Uda, M., Sanna, S., Spinhoven, P., Hartman, C.A., Sullivan, P., Realo, A., Allik, J., Heath, A.C., Pergadia, M.L., Agrawal, A., Lin, P., Grucza, R., Nutile, T., Ciullo, M., Rujescu, D., Giegling, I., Konte, B., Widen, E., Cousminer, D.L., Eriksson, J.G., Palotie, A., Peltonen, L., Luciano, M., Tenesa, A., Davies, G., Lopez, L.M., Hansell, N.K., Medland, S.E., Ferrucci, L., Schlessinger, D., Montgomery, G.W., Wright, M.J., Aulchenko, Y.S., Janssens, A.C.J.W., Oostra, B.A., Metspalu, A., Abecasis, G.R., Deary, I.J., Räikkönen, K., Bierut, L.J., Martin, N.G., van Duijn, C.M., Boomsma, D.I., 2012. Meta-analysis of genome-wide association studies for personality. Mol. Psychiatry 17, 337–349. https://doi.org/10.1038/mp.2010.128

Graff, M., Scott, R.A., Justice, A.E., Young, K.L., Feitosa, M.F., Barata, L., Winkler, T.W., Chu, A.Y., Mahajan, A., Hadley, D., Xue, L., Workalemahu, T., Heard-Costa, N.L., den Hoed, M., Ahluwalia, T.S., Qi, Q., Ngwa, J.S., Renström, F., Quaye, L., Eicher, J.D., Hayes, J.E., Cornelis, M., Kutalik, Z., Lim, E., Luan, J., Huffman, J.E., Zhang, W., Zhao, W., Griffin, P.J., Haller, T., Ahmad, S., Marques-Vidal, P.M., Bien, S., Yengo, L., Teumer, A., Smith, A.V., Kumari, M., Harder, M.N., Justesen, J.M., Kleber, M.E., Hollensted, M., Lohman, K., Rivera, N.V., Whitfield, J.B., Zhao, J.H., Stringham, H.M., Lyytikäinen, L.-P., Huppertz, C., Willemsen, G., Peyrot, W.J., Wu, Y., Kristiansson, K., Demirkan, A., Fornage, M., Hassinen, M., Bielak, L.F., Cadby, G., Tanaka, T., Mägi, R., van der Most, P.J., Jackson, A.U., Bragg-Gresham, J.L., Vitart, V., Marten, J., Navarro, P., Bellis, C., Pasko, D., Johansson, Å., Snitker, S., Cheng, Y.-C., Eriksson, J., Lim, U., Aadahl, M., Adair, L.S., Amin, N., Balkau, B., Auvinen, J., Beilby, J., Bergman, R.N., Bergmann, S., Bertoni, A.G., Blangero, J., Bonnefond, A., Bonnycastle, L.L., Borja, J.B., Brage, S., Busonero, F., Buyske, S., Campbell, H., Chines, P.S., Collins, F.S., Corre, T., Smith, G.D., Delgado, G.E., Dueker, N., Dörr, M., Ebeling, T., Eiriksdottir, G., Esko, T., Faul, J.D., Fu, M., Færch, K., Gieger, C., Gläser, S., Gong, J., Gordon-Larsen, P., Grallert, H., Grammer, T.B., Grarup, N., van Grootheest, G., Harald, K., Hastie, N.D., Havulinna, A.S., Hernandez, D., Hindorff, L., Hocking, L.J., Holmens, O.L., Holzapfel, C., Hottenga, J.J., Huang, J., Huang, T., Hui, J., Huth, C., Hutri-Kähönen, N., James, A.L., Jansson, J.-O., Jhun, M.A., Juonala, M., Kinnunen, L., Koistinen, H.A., Kolcic, I., Komulainen, P., Kuusisto, J., Kvaløy, K., Kähönen, M., Lakka, T.A., Launer, L.J., Lehne, B., Lindgren, C.M., Lorentzon, M., Luben, R., Marre, M., Milaneschi, Y., Monda, K.L., Montgomery, G.W., De Moor, M.H.M., Mulas, A., Müller-Nurasyid, M., Musk, A.W., Männikkö, R., Männistö, S., Narisu, N., Nauck, M., Nettleton, J.A., Nolte, I.M., Oldehinkel, A.J., Olden, M., Ong, K.K., Padmanabhan, S., Paternoster, L., Perez, J., Perola, M., Peters, A., Peters, U., Peyser, P.A., Prokopenko, I., Puolijoki, H., Raitakari, O.T., Rankinen, T., Rasmussen-Torvik, L.J., Rawal, R., Ridker, P.M., Rose, L.M., Rudan, I., Sarti, C., Sarzynski, M.A., Savonen, K., Scott, W.R., Sanna, S., Shuldiner, A.R., Sidney, S., Silbernagel, G., Smith, B.H., Smith, J.A., Snieder, H., Stančáková, A., Sternfeld, B., Swift, A.J., Tammelin, T., Tan, S.-T., Thorand, B., Thuillier, D., Vandenput, L., Vestergaard, H., van Vliet-Ostaptchouk, J.V., Vohl, M.-C., Völker, U., Waeber, G., Walker, M., Wild, S., Wong, A., Wright, A.F., Zillikens, M.C., Zubair, N., Haiman, C.A., Lemarchand, L., Gyllensten, U., Ohlsson, C., Hofman, A., Rivadeneira, F., Uitterlinden, A.G., Pérusse, L., Wilson, J.F., Hayward, C., Polasek, O., Cucca, F., Hveem, K., Hartman, C.A., Tönjes, A., Bandinelli, S., Palmer, L.J., Kardia, S.L.R., Rauramaa, R., Sørensen, T.I.A., Tuomilehto, J., Salomaa, V., Penninx, B.W.J.H., de Geus, E.J.C., Boomsma, D.I., Lehtimäki, T., Mangino, M., Laakso, M., Bouchard, C., Martin, N.G., Kuh, D., Liu, Y., Linneberg, A., März, W., Strauch, K., Kivimäki, M., Harris, T.B., Gudnason, V., Völzke, H., Qi, L., Järvelin, M.-R., Chambers, J.C., Kooner, J.S., Froguel, P., Kooperberg, C., Vollenweider, P., Hallmans, G., Hansen, T., Pedersen, O., Metspalu, A., Wareham, N.J., Langenberg, C., Weir, D.R., Porteous, D.J., Boerwinkle, E., Chasman, D.I., CHARGE Consortium, EPIC-InterAct Consortium, PAGE Consortium, Abecasis, G.R., Barroso, I., McCarthy, M.I., Frayling, T.M., O’Connell, J.R., van Duijn, C.M., Boehnke, M., Heid, I.M., Mohlke, K.L., Strachan, D.P., Fox, C.S., Liu, C.-T., Hirschhorn, J.N., Klein, R.J., Johnson, A.D., Borecki, I.B., Franks, P.W., North, K.E., Cupples, L.A., Loos, R.J.F., Kilpeläinen, T.O., 2017. Genome-wide physical activity interactions in adiposity - A meta-analysis of 200,452 adults. PLoS Genet. 13, e1006528. https://doi.org/10.1371/journal.pgen.1006528

Harris, S.E., Hagenaars, S.P., Davies, G., David Hill, W., Liewald, D.C.M., Ritchie, S.J., Marioni, R.E., METASTROKE Consortium, International Consortium for Blood Pressure Genome-Wide Association Studies, CHARGE Consortium Aging and Longevity Group, CHARGE Consortium Cognitive Group, Sudlow, C.L.M., Wardlaw, J.M., McIntosh, A.M., Gale, C.R., Deary, I.J., 2016. Molecular genetic contributions to self-rated health. Int. J. Epidemiol. https://doi.org/10.1093/ije/dyw219

Hill, W.D., Hagenaars, S.P., Marioni, R.E., Harris, S.E., Liewald, D.C.M., Davies, G., Okbay, A., McIntosh, A.M., Gale, C.R., Deary, I.J., 2016. Molecular Genetic Contributions to Social Deprivation and Household Income in UK Biobank. Curr. Biol. CB 26, 3083–3089. https://doi.org/10.1016/j.cub.2016.09.035

Hou, L., Bergen, S.E., Akula, N., Song, J., Hultman, C.M., Landén, M., Adli, M., Alda, M., Ardau, R., Arias, B., Aubry, J.-M., Backlund, L., Badner, J.A., Barrett, T.B., Bauer, M., Baune, B.T., Bellivier, F., Benabarre, A., Bengesser, S., Berrettini, W.H., Bhattacharjee, A.K., Biernacka, J.M., Birner, A., Bloss, C.S., Brichant-Petitjean, C., Bui, E.T., Byerley, W., Cervantes, P., Chillotti, C., Cichon, S., Colom, F., Coryell, W., Craig, D.W., Cruceanu, C., Czerski, P.M., Davis, T., Dayer, A., Degenhardt, F., Del Zompo, M., DePaulo, J.R., Edenberg, H.J., Étain, B., Falkai, P., Foroud, T., Forstner, A.J., Frisén, L., Frye, M.A., Fullerton, J.M., Gard, S., Garnham, J.S., Gershon, E.S., Goes, F.S., Greenwood, T.A., Grigoroiu-Serbanescu, M., Hauser, J., Heilbronner, U., Heilmann-Heimbach, S., Herms, S., Hipolito, M., Hitturlingappa, S., Hoffmann, P., Hofmann, A., Jamain, S., Jiménez, E., Kahn, J.-P., Kassem, L., Kelsoe, J.R., Kittel-Schneider, S., Kliwicki, S., Koller, D.L., König, B., Lackner, N., Laje, G., Lang, M., Lavebratt, C., Lawson, W.B., Leboyer, M., Leckband, S.G., Liu, C., Maaser, A., Mahon, P.B., Maier, W., Maj, M., Manchia, M., Martinsson, L., McCarthy, M.J., McElroy, S.L., McInnis, M.G., McKinney, R., Mitchell, P.B., Mitjans, M., Mondimore, F.M., Monteleone, P., Mühleisen, T.W., Nievergelt, C.M., Nöthen, M.M., Novák, T., Nurnberger, J.I., Nwulia, E.A., Ösby, U., Pfennig, A., Potash, J.B., Propping, P., Reif, A., Reininghaus, E., Rice, J., Rietschel, M., Rouleau, G.A., Rybakowski, J.K., Schalling, M., Scheftner, W.A., Schofield, P.R., Schork, N.J., Schulze, T.G., Schumacher, J., Schweizer, B.W., Severino, G., Shekhtman, T., Shilling, P.D., Simhandl, C., Slaney, C.M., Smith, E.N., Squassina, A., Stamm, T., Stopkova, P., Streit, F., Strohmaier, J., Szelinger, S., Tighe, S.K., Tortorella, A., Turecki, G., Vieta, E., Volkert, J., Witt, S.H., Wright, A., Zandi, P.P., Zhang, P., Zollner, S., McMahon, F.J., 2016. Genome-wide association study of 40,000 individuals identifies two novel loci associated with bipolar disorder. Hum. Mol. Genet. 25, 3383–3394. https://doi.org/10.1093/hmg/ddw181

Hu, Y., Shmygelska, A., Tran, D., Eriksson, N., Tung, J.Y., Hinds, D.A., 2016. GWAS of 89,283 individuals identifies genetic variants associated with self-reporting of being a morning person. Nat. Commun. 7, 10448. https://doi.org/10.1038/ncomms10448

Liu, J.Z., van Sommeren, S., Huang, H., Ng, S.C., Alberts, R., Takahashi, A., Ripke, S., Lee, J.C., Jostins, L., Shah, T., Abedian, S., Cheon, J.H., Cho, J., Daryani, N.E., Franke, L., Fuyuno, Y., Hart, A., Juyal, R.C., Juyal, G., Kim, W.H., Morris, A.P., Poustchi, H., Newman, W.G., Midha, V., Orchard, T.R., Vahedi, H., Sood, A., Sung, J.J.Y., Malekzadeh, R., Westra, H.-J., Yamazaki, K., Yang, S.-K., International Multiple Sclerosis Genetics Consortium, International IBD Genetics Consortium, Barrett, J.C., Franke, A., Alizadeh, B.Z., Parkes, M., B K, T., Daly, M.J., Kubo, M., Anderson, C.A., Weersma, R.K., 2015. Association analyses identify 38 susceptibility loci for inflammatory bowel disease and highlight shared genetic risk across populations. Nat. Genet. 47, 979–986. https://doi.org/10.1038/ng.3359

Major Depressive Disorder Working Group of the Psychiatric GWAS Consortium, Ripke, S., Wray, N.R., Lewis, C.M., Hamilton, S.P., Weissman, M.M., Breen, G., Byrne, E.M., Blackwood, D.H.R., Boomsma, D.I., Cichon, S., Heath, A.C., Holsboer, F., Lucae, S., Madden, P.A.F., Martin, N.G., McGuffin, P., Muglia, P., Noethen, M.M., Penninx, B.P., Pergadia, M.L., Potash, J.B., Rietschel, M., Lin, D., Müller-Myhsok, B., Shi, J., Steinberg, S., Grabe, H.J., Lichtenstein, P., Magnusson, P., Perlis, R.H., Preisig, M., Smoller, J.W., Stefansson, K., Uher, R., Kutalik, Z., Tansey, K.E., Teumer, A., Viktorin, A., Barnes, M.R., Bettecken, T., Binder, E.B., Breuer, R., Castro, V.M., Churchill, S.E., Coryell, W.H., Craddock, N., Craig, I.W., Czamara, D., De Geus, E.J., Degenhardt, F., Farmer, A.E., Fava, M., Frank, J., Gainer, V.S., Gallagher, P.J., Gordon, S.D., Goryachev, S., Gross, M., Guipponi, M., Henders, A.K., Herms, S., Hickie, I.B., Hoefels, S., Hoogendijk, W., Hottenga, J.J., Iosifescu, D.V., Ising, M., Jones, I., Jones, L., Jung-Ying, T., Knowles, J.A., Kohane, I.S., Kohli, M.A., Korszun, A., Landen, M., Lawson, W.B., Lewis, G., Macintyre, D., Maier, W., Mattheisen, M., McGrath, P.J., McIntosh, A., McLean, A., Middeldorp, C.M., Middleton, L., Montgomery, G.M., Murphy, S.N., Nauck, M., Nolen, W.A., Nyholt, D.R., O’Donovan, M., Oskarsson, H., Pedersen, N., Scheftner, W.A., Schulz, A., Schulze, T.G., Shyn, S.I., Sigurdsson, E., Slager, S.L., Smit, J.H., Stefansson, H., Steffens, M., Thorgeirsson, T., Tozzi, F., Treutlein, J., Uhr, M., van den Oord, E.J.C.G., Van Grootheest, G., Völzke, H., Weilburg, J.B., Willemsen, G., Zitman, F.G., Neale, B., Daly, M., Levinson, D.F., Sullivan, P.F., 2013. A mega-analysis of genome-wide association studies for major depressive disorder. Mol. Psychiatry 18, 497–511. https://doi.org/10.1038/mp.2012.21

Nikpay, M., Goel, A., Won, H.-H., Hall, L.M., Willenborg, C., Kanoni, S., Saleheen, D., Kyriakou, T., Nelson, C.P., Hopewell, J.C., Webb, T.R., Zeng, L., Dehghan, A., Alver, M., Armasu, S.M., Auro, K., Bjonnes, A., Chasman, D.I., Chen, S., Ford, I., Franceschini, N., Gieger, C., Grace, C., Gustafsson, S., Huang, Jie, Hwang, S.-J., Kim, Y.K., Kleber, M.E., Lau, K.W., Lu, X., Lu, Y., Lyytikäinen, L.-P., Mihailov, E., Morrison, A.C., Pervjakova, N., Qu, L., Rose, L.M., Salfati, E., Saxena, R., Scholz, M., Smith, A.V., Tikkanen, E., Uitterlinden, A., Yang, X., Zhang, W., Zhao, W., de Andrade, M., de Vries, P.S., van Zuydam, N.R., Anand, S.S., Bertram, L., Beutner, F., Dedoussis, G., Frossard, P., Gauguier, D., Goodall, A.H., Gottesman, O., Haber, M., Han, B.-G., Huang, Jianfeng, Jalilzadeh, S., Kessler, T., König, I.R., Lannfelt, L., Lieb, W., Lind, L., Lindgren, C.M., Lokki, M.-L., Magnusson, P.K., Mallick, N.H., Mehra, N., Meitinger, T., Memon, F.-U.-R., Morris, A.P., Nieminen, M.S., Pedersen, N.L., Peters, A., Rallidis, L.S., Rasheed, A., Samuel, M., Shah, S.H., Sinisalo, J., Stirrups, K.E., Trompet, S., Wang, L., Zaman, K.S., Ardissino, D., Boerwinkle, E., Borecki, I.B., Bottinger, E.P., Buring, J.E., Chambers, J.C., Collins, R., Cupples, L.A., Danesh, J., Demuth, I., Elosua, R., Epstein, S.E., Esko, T., Feitosa, M.F., Franco, O.H., Franzosi, M.G., Granger, C.B., Gu, D., Gudnason, V., Hall, A.S., Hamsten, A., Harris, T.B., Hazen, S.L., Hengstenberg, C., Hofman, A., Ingelsson, E., Iribarren, C., Jukema, J.W., Karhunen, P.J., Kim, B.-J., Kooner, J.S., Kullo, I.J., Lehtimäki, T., Loos, R.J.F., Melander, O., Metspalu, A., März, W., Palmer, C.N., Perola, M., Quertermous, T., Rader, D.J., Ridker, P.M., Ripatti, S., Roberts, R., Salomaa, V., Sanghera, D.K., Schwartz, S.M., Seedorf, U., Stewart, A.F., Stott, D.J., Thiery, J., Zalloua, P.A., O’Donnell, C.J., Reilly, M.P., Assimes, T.L., Thompson, J.R., Erdmann, J., Clarke, R., Watkins, H., Kathiresan, S., McPherson, R., Deloukas, P., Schunkert, H., Samani, N.J., Farrall, M., 2015. A comprehensive 1,000 Genomes-based genome-wide association meta-analysis of coronary artery disease. Nat. Genet. 47, 1121–1130. https://doi.org/10.1038/ng.3396

Okbay, A., Baselmans, B.M.L., De Neve, J.-E., Turley, P., Nivard, M.G., Fontana, M.A., Meddens, S.F.W., Linnér, R.K., Rietveld, C.A., Derringer, J., Gratten, J., Lee, J.J., Liu, J.Z., de Vlaming, R., Ahluwalia, T.S., Buchwald, J., Cavadino, A., Frazier-Wood, A.C., Davies, G., Furlotte, N.A., Garfield, V., Geisel, M.H., Gonzalez, J.R., Haitjema, S., Karlsson, R., van der Laan, S.W., Ladwig, K.-H., Lahti, J., van der Lee, S.J., Miller, M.B., Lind, P.A., Liu, T., Matteson, L., Mihailov, E., Minica, C.C., Nolte, I.M., Mook-Kanamori, D.O., van der Most, P.J., Oldmeadow, C., Qian, Y., Raitakari, O., Rawal, R., Realo, A., Rueedi, R., Schmidt, B., Smith, A.V., Stergiakouli, E., Tanaka, T., Taylor, K., Thorleifsson, G., Wedenoja, J., Wellmann, J., Westra, H.-J., Willems, S.M., Zhao, W., Amin, N., Bakshi, A., Bergmann, S., Bjornsdottir, G., Boyle, P.A., Cherney, S., Cox, S.R., Davis, O.S.P., Ding, J., Direk, N., Eibich, P., Emeny, R.T., Fatemifar, G., Faul, J.D., Ferrucci, L., Forstner, A.J., Gieger, C., Gupta, R., Harris, T.B., Harris, J.M., Holliday, E.G., Hottenga, J.-J., De Jager, P.L., Kaakinen, M.A., Kajantie, E., Karhunen, V., Kolcic, I., Kumari, M., Launer, L.J., Franke, L., Li-Gao, R., Liewald, D.C., Koini, M., Loukola, A., Marques-Vidal, P., Montgomery, G.W., Mosing, M.A., Paternoster, L., Pattie, A., Petrovic, K.E., Pulkki-Råback, L., Quaye, L., Räikkönen, K., Rudan, I., Scott, R.J., Smith, J.A., Sutin, A.R., Trzaskowski, M., Vinkhuyzen, A.E., Yu, L., Zabaneh, D., Attia, J.R., Bennett, D.A., Berger, K., Bertram, L., Boomsma, D.I., Snieder, H., Chang, S.-C., Cucca, F., Deary, I.J., van Duijn, C.M., Eriksson, J.G., Bültmann, U., de Geus, E.J.C., Groenen, P.J.F., Gudnason, V., Hansen, T., Hartman, C.A., Haworth, C.M.A., Hayward, C., Heath, A.C., Hinds, D.A., Hyppönen, E., Iacono, W.G., Järvelin, M.-R., Jöckel, K.-H., Kaprio, J., Kardia, S.L.R., Keltikangas-Järvinen, L., Kraft, P., Kubzansky, L.D., Lehtimäki, T., Magnusson, P.K.E., Martin, N.G., McGue, M., Metspalu, A., Mills, M., de Mutsert, R., Oldehinkel, A.J., Pasterkamp, G., Pedersen, N.L., Plomin, R., Polasek, O., Power, C., Rich, S.S., Rosendaal, F.R., den Ruijter, H.M., Schlessinger, D., Schmidt, H., Svento, R., Schmidt, R., Alizadeh, B.Z., Sørensen, T.I.A., Spector, T.D., Starr, J.M., Stefansson, K., Steptoe, A., Terracciano, A., Thorsteinsdottir, U., Thurik, A.R., Timpson, N.J., Tiemeier, H., Uitterlinden, A.G., Vollenweider, P., Wagner, G.G., Weir, D.R., Yang, J., Conley, D.C., Smith, G.D., Hofman, A., Johannesson, M., Laibson, D.I., Medland, S.E., Meyer, M.N., Pickrell, J.K., Esko, T., Krueger, R.F., Beauchamp, J.P., Koellinger, P.D., Benjamin, D.J., Bartels, M., Cesarini, D., 2016a. Genetic variants associated with subjective well-being, depressive symptoms and neuroticism identified through genome-wide analyses. Nat. Genet. 48, 624–633. https://doi.org/10.1038/ng.3552

Okbay, A., Beauchamp, J.P., Fontana, M.A., Lee, J.J., Pers, T.H., Rietveld, C.A., Turley, P., Chen, G.-B., Emilsson, V., Meddens, S.F.W., Oskarsson, S., Pickrell, J.K., Thom, K., Timshel, P., de Vlaming, R., Abdellaoui, A., Ahluwalia, T.S., Bacelis, J., Baumbach, C., Bjornsdottir, G., Brandsma, J.H., Pina Concas, M., Derringer, J., Furlotte, N.A., Galesloot, T.E., Girotto, G., Gupta, R., Hall, L.M., Harris, S.E., Hofer, E., Horikoshi, M., Huffman, J.E., Kaasik, K., Kalafati, I.P., Karlsson, R., Kong, A., Lahti, J., van der Lee, S.J., deLeeuw, C., Lind, P.A., Lindgren, K.-O., Liu, T., Mangino, M., Marten, J., Mihailov, E., Miller, M.B., van der Most, P.J., Oldmeadow, C., Payton, A., Pervjakova, N., Peyrot, W.J., Qian, Y., Raitakari, O., Rueedi, R., Salvi, E., Schmidt, B., Schraut, K.E., Shi, J., Smith, A.V., Poot, R.A., St Pourcain, B., Teumer, A., Thorleifsson, G., Verweij, N., Vuckovic, D., Wellmann, J., Westra, H.-J., Yang, Jingyun, Zhao, W., Zhu, Z., Alizadeh, B.Z., Amin, N., Bakshi, A., Baumeister, S.E., Biino, G., Bønnelykke, K., Boyle, P.A., Campbell, H., Cappuccio, F.P., Davies, G., De Neve, J.-E., Deloukas, P., Demuth, I., Ding, J., Eibich, P., Eisele, L., Eklund, N., Evans, D.M., Faul, J.D., Feitosa, M.F., Forstner, A.J., Gandin, I., Gunnarsson, B., Halldórsson, B.V., Harris, T.B., Heath, A.C., Hocking, L.J., Holliday, E.G., Homuth, G., Horan, M.A., Hottenga, J.-J., de Jager, P.L., Joshi, P.K., Jugessur, A., Kaakinen, M.A., Kähönen, M., Kanoni, S., Keltigangas-Järvinen, L., Kiemeney, L.A.L.M., Kolcic, I., Koskinen, S., Kraja, A.T., Kroh, M., Kutalik, Z., Latvala, A., Launer, L.J., Lebreton, M.P., Levinson, D.F., Lichtenstein, P., Lichtner, P., Liewald, D.C.M., LifeLines Cohort Study, Loukola, A., Madden, P.A., Mägi, R., Mäki-Opas, T., Marioni, R.E., Marques-Vidal, P., Meddens, G.A., McMahon, G., Meisinger, C., Meitinger, T., Milaneschi, Y., Milani, L., Montgomery, G.W., Myhre, R., Nelson, C.P., Nyholt, D.R., Ollier, W.E.R., Palotie, A., Paternoster, L., Pedersen, N.L., Petrovic, K.E., Porteous, D.J., Räikkönen, K., Ring, S.M., Robino, A., Rostapshova, O., Rudan, I., Rustichini, A., Salomaa, V., Sanders, A.R., Sarin, A.-P., Schmidt, H., Scott, R.J., Smith, B.H., Smith, J.A., Staessen, J.A., Steinhagen-Thiessen, E., Strauch, K., Terracciano, A., Tobin, M.D., Ulivi, S., Vaccargiu, S., Quaye, L., van Rooij, F.J.A., Venturini, C., Vinkhuyzen, A.A.E., Völker, U., Völzke, H., Vonk, J.M., Vozzi, D., Waage, J., Ware, E.B., Willemsen, G., Attia, J.R., Bennett, D.A., Berger, K., Bertram, L., Bisgaard, H., Boomsma, D.I., Borecki, I.B., Bültmann, U., Chabris, C.F., Cucca, F., Cusi, D., Deary, I.J., Dedoussis, G.V., van Duijn, C.M., Eriksson, J.G., Franke, B., Franke, L., Gasparini, P., Gejman, P.V., Gieger, C., Grabe, H.-J., Gratten, J., Groenen, P.J.F., Gudnason, V., van der Harst, P., Hayward, C., Hinds, D.A., Hoffmann, W., Hyppönen, E., Iacono, W.G., Jacobsson, B., Järvelin, M.-R., Jöckel, K.-H., Kaprio, J., Kardia, S.L.R., Lehtimäki, T., Lehrer, S.F., Magnusson, P.K.E., Martin, N.G., McGue, M., Metspalu, A., Pendleton, N., Penninx, B.W.J.H., Perola, M., Pirastu, N., Pirastu, M., Polasek, O., Posthuma, D., Power, C., Province, M.A., Samani, N.J., Schlessinger, D., Schmidt, R., Sørensen, T.I.A., Spector, T.D., Stefansson, K., Thorsteinsdottir, U., Thurik, A.R., Timpson, N.J., Tiemeier, H., Tung, J.Y., Uitterlinden, A.G., Vitart, V., Vollenweider, P., Weir, D.R., Wilson, J.F., Wright, A.F., Conley, D.C., Krueger, R.F., Davey Smith, G., Hofman, A., Laibson, D.I., Medland, S.E., Meyer, M.N., Yang, Jian, Johannesson, M., Visscher, P.M., Esko, T., Koellinger, P.D., Cesarini, D., Benjamin, D.J., 2016b. Genome-wide association study identifies 74 loci associated with educational attainment. Nature 533, 539–542. https://doi.org/10.1038/nature17671

Psychiatric GWAS Consortium Bipolar Disorder Working Group, 2011. Large-scale genome-wide association analysis of bipolar disorder identifies a new susceptibility locus near ODZ4. Nat. Genet. 43, 977–983. https://doi.org/10.1038/ng.943

Sherva, R., Wang, Q., Kranzler, H., Zhao, H., Koesterer, R., Herman, A., Farrer, L.A., Gelernter, J., 2016. Genome-wide Association Study of Cannabis Dependence Severity, Novel Risk Variants, and Shared Genetic Risks. JAMA Psychiatry 73, 472–480. https://doi.org/10.1001/jamapsychiatry.2016.0036

Tobacco and Genetics Consortium, 2010. Genome-wide meta-analyses identify multiple loci associated with smoking behavior. Nat. Genet. 42, 441–447. https://doi.org/10.1038/ng.571
